# Insights into the action of phylogenetically diverse microbial expansins on the structure of cellulose microfibrils

**DOI:** 10.1186/s13068-024-02500-w

**Published:** 2024-04-23

**Authors:** Majid Haddad Momeni, Aleksi Zitting, Vilma Jäämuru, Rosaliina Turunen, Paavo Penttilä, Garry W. Buchko, Salla Hiltunen, Natalia Maiorova, Anu Koivula, Janak Sapkota, Kaisa Marjamaa, Emma R. Master

**Affiliations:** 1 Department of Bioproducts and Biosystems, Aalto University, Kemistintie 1, 02150 Espoo, Finland; 2 Earth and Biological Sciences Directorate, Pacific Northwest National Laboratory, Richland, WA 99354 USA; 3 School of Molecular Biosciences, Washington State University, Pullman, WA 99164 USA; 4 NE Research Center, UPM Pulp Research and Innovations, 53200 Lappeenranta, Finland; 5 VTT Technical Research Centre of Finland Ltd, P.O. Box 1000, 02044-VTT Espoo, Finland; 6 Department of Chemical Engineering and Applied Chemistry, University of Toronto, 200 College Street, Toronto, ON M5S 3E5 Canada

**Keywords:** Expansin, Non-lytic, Cellulose, Carbohydrate-active enzymes, Defibrillation

## Abstract

**Background:**

Microbial expansins (EXLXs) are non-lytic proteins homologous to plant expansins involved in plant cell wall formation. Due to their non-lytic cell wall loosening properties and potential to disaggregate cellulosic structures, there is considerable interest in exploring the ability of microbial expansins (EXLX) to assist the processing of cellulosic biomass for broader biotechnological applications. Herein, EXLXs with different modular structure and from diverse phylogenetic origin were compared in terms of ability to bind cellulosic, xylosic, and chitinous substrates, to structurally modify cellulosic fibrils, and to boost enzymatic deconstruction of hardwood pulp.

**Results:**

Five heterogeneously produced EXLXs (*Clavibacter michiganensis; Cmi*EXLX2, *Dickeya aquatica; Daq*EXLX1, *Xanthomonas sacchari; Xsa*EXLX1, *Nothophytophthora sp.; Nsp*EXLX1 and *Phytophthora cactorum; Pca*EXLX1) were shown to bind xylan and hardwood pulp at pH 5.5 and *Cmi*EXLX2 (harboring a family-2 carbohydrate-binding module) also bound well to crystalline cellulose. Small-angle X-ray scattering revealed a 20–25% increase in interfibrillar distance between neighboring cellulose microfibrils following treatment with *Cmi*EXLX2, *Daq*EXLX1, or *Nsp*EXLX1. Correspondingly, combining xylanase with *Cmi*EXLX2 and *Daq*EXLX1 increased product yield from hardwood pulp by ~ 25%, while supplementing the *Tr*AA9A LPMO from *Trichoderma reesei* with *Cmi*EXLX2, *Daq*EXLX1, and *Nsp*EXLX1 increased total product yield by over 35%.

**Conclusion:**

This direct comparison of diverse EXLXs revealed consistent impacts on interfibrillar spacing of cellulose microfibers and performance of carbohydrate-active enzymes predicted to act on fiber surfaces. These findings uncover new possibilities to employ EXLXs in the creation of value-added materials from cellulosic biomass.

**Supplementary Information:**

The online version contains supplementary material available at 10.1186/s13068-024-02500-w.

## Background

Expansins are non-lytic proteins first discovered in plants where they reportedly disrupt non-covalent bonds at load bearing junctions between cellulose microfibrils and matrix biopolymers within plant cell walls, promoting plant cell wall loosening [1–4]. Homologous proteins have also been identified among many bacterial, fungal, and amoebozoal organisms [5–8] where they are implicated in plant pathogenesis [9, 10], plant cell wall (i.e., lignocellulose) deconstruction [11, 12], and microbial cell wall development [13]. Due to their non-lytic cell wall loosening properties and potential to disaggregate cellulosic structures [14–16], there is considerable interest in exploring the use of microbial expansins to assist the processing of cellulosic biomass for broader application in materials, fuels, and chemicals.

In plants, expansins are grouped in two major gene families, α-expansins (EXPA) and β-expansins (EXPB), and 2 minor gene families containing expansin-like genes of uncertain function (EXLA and EXLB) [17]. In non-plants, genes homologous to expansins are designated EXLX. To date, eleven plant expansin and ten bacterial expansin structures have been solved [18], including multiple structures of EXPB1 (aka Zea m1) from maize [4] and *Bs*EXLX1 from *Bacillus subtilis* [13]. Both plant and microbial expansins are composed of two tightly packed domains: D1 and D2 [13]. The N-terminal D1 domain adopts a double-ψ β-barrel fold (DPBB) and is structurally homologous to the glycoside hydrolase family 45 (GH45) except for the absence of the catalytic residue necessary for hydrolysis. Site-directed mutagenesis of the D1 domain of *Bs*EXLX1 show that D71, Y73, and D82 are essential for expansin function via an unknown mechanism [19]. The C-terminal D2 domain adopts a β-sandwich with an Ig-like fold and has been assigned to the Type-A carbohydrate-binding module family 63 (CBM63). *Bs*EXLX1 crystal structures with cellohexaose show the D2 domain binds β-(1 → 4)-linked glucans via CH–π interactions between the cellohexaose and three linearly arranged aromatic amino acids (W125, W126, and Y157); lysine K119 further stabilizes this interaction through hydrogen bonding with the G5 sugar [18, 19].

Besides expansin-like proteins, microbial expansin-related proteins include loosenins and ceratoplatanins that lack the C-terminal D2 domain [20, 21], fungal swollenins that extend the core expansin structure with an N-terminal CBM1 domain and a putative linker known as a fibronectin III domain [22], and bacterial proteins that extend the core structure with additional CBM and GH domains (e.g., CBM2 and GH5, respectively) [5, 7]. Whereas plant and microbial expansins lack lytic activity, certain swollenins display low levels of hydrolytic activity toward β-glucans, carboxymethyl cellulose, and cello-oligosaccharides despite lacking a bonafide GH domain [23].

Given the difficulties to heterologously express plant expansins, the functional characterization of expansins has largely focused on microbial homologues with potential to disrupt cellulose fiber networks and aid in lignocellulose processing. Complementary biophysical methods have been used to study microbial expansins, including measures of cellulose strength, crystallinity, and morphology after expansin treatment. For example, *Tr*SWO1, a swollenin from *Trichoderma reesei*, was shown to reduce the tensile strength of filter paper while inducing swelling of mercerized cotton fibers after sonication [22]. Filter paper weakening has since been reported for several bacterial expansins (*Bs*EXLX1[19], *Hc*EXLX2 from *Hahella chejuensis* [24], *Pc*Exl1 from *Pectobacterium carotovorum* [10]) and expansin-related proteins (fungal loosenins from *Bjerkandera adusta* [20] and *Phanerochaete carnosa* [21]). Using X-ray diffraction, Jager et al. 2011 [25] revealed that *Tr*SWO1 can reduce the crystallinity of multiple cellulose preparations (e.g., filter paper, Avicel, alpha cellulose) with small percentual changes; similarly, Duan et al. 2018 [26] revealed that *Bs*EXLX1 can reduce the crystallinity of Avicel by 6% as shown by increasing nitrogen adsorption. On the other hand, a change in crystallinity of Avicel was not observed using wide-angle X-ray scattering (WAXS) with *Tr*SWO1 [27] or SWO2 from *Trichoderma pseudokoningii* S38 [28]; moreover, *Tr*SWO1 treatment of mercerized cotton was shown to promote adsorption of CBM2 which preferentially binds crystalline cellulose [29]. Similarly, studies using scanning electron microscopy and atomic force microscopy to analyze cellulosic material treated with *Tr*SWO1 reveal smooth fiber surfaces lacking evidence of amorphogenesis [27]. Together, these studies laid a foundation of complementary methods for expansin characterization, and at the same time, revealed the varying effects of different microbial expansins along with impacts of substrate on the observed protein action.

Expansins that increase cellulose fiber accessibility through fibrillation or by reducing cellulose crystallinity could enhance the enzymatic deconstruction of lignocellulosic substrates [14]. In nature, lignocellulose is decomposed by microorganisms that secrete a wide array of carbohydrate-active enzymes as classified by the Carbohydrate-Active enZyme (CAZy) database [30]. The synergistic action of diverse carbohydrate-active enzymes on cellulosic substrates is well-described and includes the concerted action of both hydrolytic and oxidative enzymes. The potential for microbial expansins to enhance enzymatic deconstruction of lignocellulose has been investigated using different lignocellulosic substrates [31–33]. As reviewed elsewhere [34], the measured impacts of expansins on enzymatic lignocellulose deconstruction depends on several factors, including the nature of substrate, type of pretreatment, reaction time, expansin dose, lytic-enzyme dose, and the applied enzyme system. Overall, the greatest impacts of expansins on enzymatic lignocellulose deconstruction treatments have been observed using comparatively low lytic-enzyme doses and cellulosic substrates characterized by comparatively high hemicellulose and low lignin contents [12, 32].

The current study reports the functional characterization of three bacterial and two eukaryotic expansins that belong to distinct phylogenetic clades. The recombinantly produced microbial expansins were compared in terms of their ability to bind cellulosic, xylosic, and chitinous substrates, to structurally modify cellulosic fibrils, and boost enzymatic deconstruction of hardwood pulp. To better quantify expansin-mediated cellulose fibrillation, scanning electron microscopy (SEM) studies were complemented with small-angle X-ray scattering (SAXS) to directly measure changes in interfibrillar spacing. Solution state NMR spectroscopic studies on one of the expansins identified unusual dynamics that may offer clues into the molecular mechanism expansins employ to promote plant cell wall loosening. The direct comparison of diverse microbial expansins reported herein reveals consistent impacts on interfibrillar spacing of cellulose microfibers and performance of lytic enzymes expected to act on fiber surfaces.

## Results

### Selection and production of microbial expansins

**Fig. 1:**
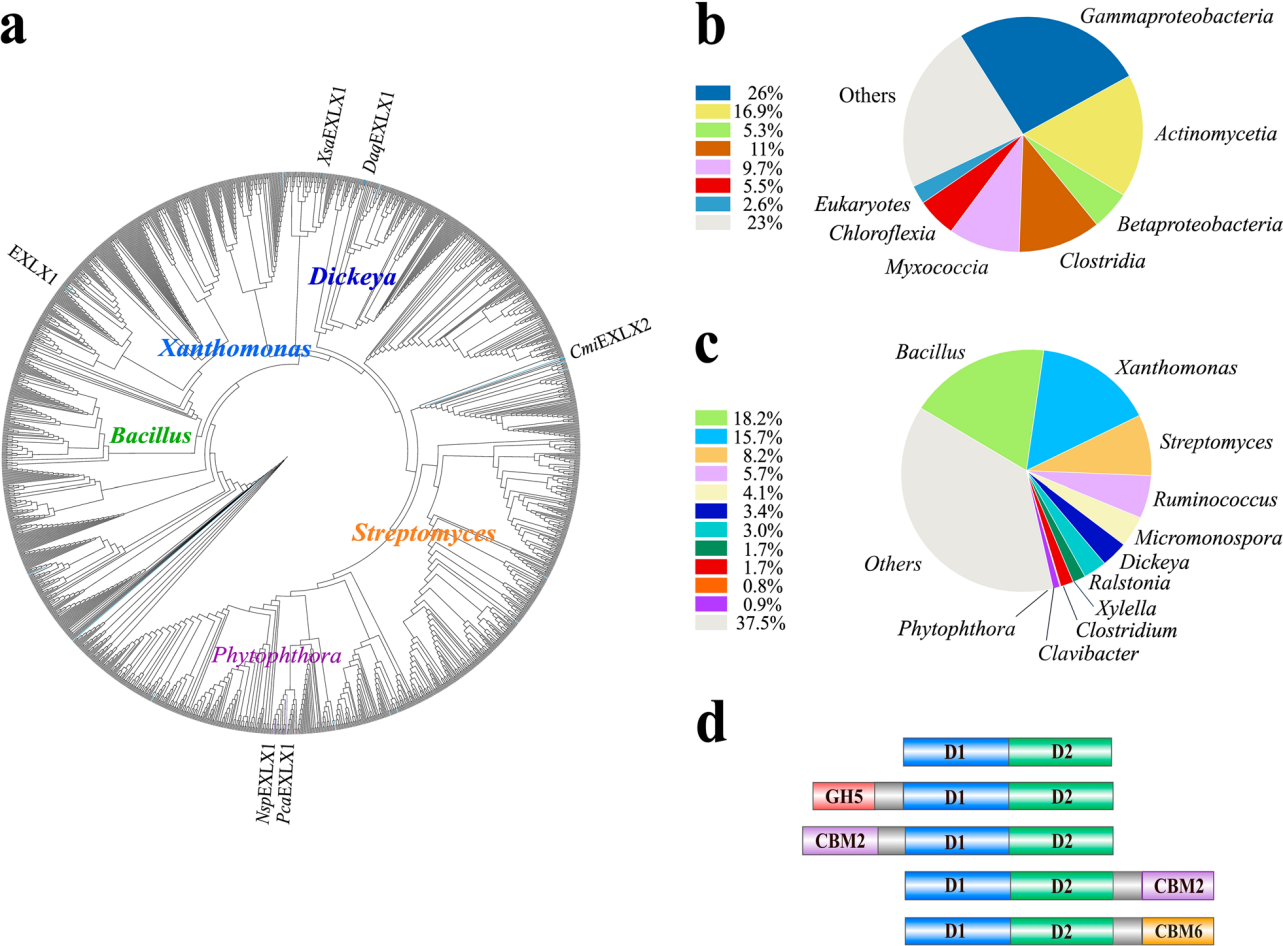
Phylogenetic analysis of microbial expansin sequences. **a** Phylogenetic tree constructed from 1575 sequences related to *Bacillus subtilis,* EXLX1. The major cluster of expansin-like proteins from bacterial and Phytophthora organisms and query expansin-like sequence from *B. subtilis* EXLX1 are highlighted on the outside of the tree. Bootstrap values were calculated with 900 iterations. **b** Microbial phyla encoding expansin-like sequences include bacterial plant pathogens (> 97%) and Eukaryotes (< 3%). **c** The distribution of dominant bacterial genera (> 99%) versus Phytophthora (< 1%) that encode expansin-like sequences. **d** The modular organization found among retrieved expansin-like sequences. The gray bar indicates the linker region among Carbohydrate-Binding Module 2 (CBM2) and the D1 or D2 domain. GH5 indicates Glycoside Hydrolase family 5

To explore the sequence diversity and phylogenetic relationship among microbial expansins, the sequence of the characterized *Bs*EXLX1 from *Bacillus subtilis* was used as a query to retrieve over 4000 protein sequences from the non-redundant database at NCBI (excluding plant-based expansin genes). The retrieved sequences originating from bacterial, oomycete, and Amoebozoa microorganisms were aligned and curated to construct a phylogenetic tree (Fig. 1a). The fungal expansin-like genes were not among the retrieved sequences due to their low similarity (< 30%) to the query. Consistent with recent phylogenetic analyses of microbial expansins [35], the analyses performed herein to guide selections for functional characterization delineated the sequences belonging to Gammaproteobacteria (26%), Actinobacteria (16.9%), Betaproteobacteria (5.3%), and *Oomycota* (~ 1%) (Fig. 1b). Distinct clades comprised sequences from *Bacillus* (18.2%), *Xanthomonas* (15.7%), *Streptomyces* (8.2%), *Dickeya* (3.4%), *Ralstonia* (3.0%), and *Xylella* (1.6%). Notably, the *Phytophthora* in the *Oomycota* phylum encodes 15 expansins which represented nearly 1% of the total retrieved sequences included in the phylogenetic analysis (Fig. 1c).

All recovered sequences contained both the D1 (DPBB) and D2 (CBM63) domains characteristic of expansins, with < 1% containing an additional domain. Appended additional domains primarily belonged to either the CBM2 or GH5 families; were appended to the N-terminal end of the protein (Fig. 1d); and originated from *Xanthomonas, Clavibacter, Dactylosporangium,* or *Mitsuaria sp. bacterial species* (Additional file 1: Table S1). Based on our sequence analyses, 16 bacterial and 5 eukaryotic genes encoding expansins were ordered as synthetic genes for recombinant protein production.

Of the 16 bacterial expansins selected for expression in *E. coli* (BL21), 8 expressed at detectable levels with yields ranging from 0.5 to 24 mg L^−1^. The highest expression yields were achieved for *Xanthomonas sacchari (Xsa*EXLX1), *Dickeya aquatica* (*Daq*EXLX1), and *Clavibacter michiganensis* (*Cmi*EXLX2) (Additional file 1: Table S2). Of the five eukaryotic expansins selected for expression in *Pichia pastoris*, *Nothophytophthora sp.* (*Nsp*EXLX1) and *Phytophthora cactorum* (*Pca*EXLX1) expressed at 50 mg L^−1^ and 41 mg L^−1^, respectively; expression levels for the other eukaryotic targets were not sufficient for characterization (Additional file 1: Table S2). Accordingly, *Xsa*EXLX1, *Daq*EXLX1, *Cmi*EXLX2, *Nsp*EXLX1, and *Pca*EXLX1 were selected for production (in shake flasks) and further functional characterization. All selected proteins retained the conserved polysaccharide binding residues present in the D2 domain of *Bs*EXLX1 (W125, W126, Y157, and K119) and the residues deemed essential for expansin function in the D1 domain (D71, Y73, and D82). The overall sequence identity between the five microbial expansins and *Bs*EXLX1 ranged from 36 to 76% (Additional file 1: Fig. S1).

### Temperature stability

The thermal transition mid-point of each purified protein was measured in 25 mM sodium acetate buffer (pH 5.5) using label‐free differential scanning fluorimetry (nanoDSF), which measures change in the intrinsic fluorescence of tryptophan and tyrosine residues present in the protein [36]. All proteins displayed T_m_ values between 52.0 °C and 72.0 °C with the maximum melting temperature observed for *Nsp*EXLX1 (Additional file 1: Fig. S2a-d). T_m_ values determined using CD spectroscopy [37], between 50.8 °C and 65.1 °C, were within the experimental error with the values determined with nanoDSF (Additional file 1: Fig. S3).

### Structural characterization by CD and NMR spectroscopy

Steady-state wavelength CD spectra were collected on the five targets to survey their solution structures. As illustrated (Additional file 1: Fig. S3), the dominant feature of the CD spectra is a maximum at ~ 205 nm or lower wavelengths that is characteristic in proteins with α-helical and β-sheet structure [38]. A broad single minimum at ~ 215 nm for *Cmi*EXLX2 and *Nsp*EXLX1, the 2 eukaryotic targets, suggests β-sheet secondary structure is prominent in these two proteins. On the other hand, the absence of major wavelength minima between 208 and 235 nm for the other three proteins suggests these targets contain a significant population of random coil. To further explore the structural features of the bacterial expansins, both *Daq*EXLX1 and *Xsa*EXLX1 were ^15^N-labeled and ^1^H-^15^N HSQC spectra collected. As illustrated for both proteins (Additional file 1: Fig. S4), their ^1^H-^15^N HSQC spectra featured wide chemical shift dispersion of the amide resonances in both the nitrogen and proton dimension, a feature characteristic of a structured protein [39]. Due to the low yields for *Daq*EXLX1 in minimal media, *Xsa*EXLX1 alone was ^13^C- and ^15^N -labeled for further NMR analyses. It soon became apparent from the analysis of the three-dimensional NMR backbone assignment data for *Xsa*EXLX1 (23.4 kDa) that the protein would need deuteration of the non-exchangeable protons to make the chemical shift assignments. An unexpected consequence of perdeuteration of *Xsa*EXLX1, where the only difference in sample preparation was the use of 98% D_2_O instead of H_2_O in the media, was the disappearance of ~ 40 amide resonances in its ^1^H- ^15^N HSQC spectrum (Additional file 1: Fig. S5). All efforts to convert the ^2^H-, ^13^C-, ^15^N-*Xsa*EXLX1 ^1^H-^15^N HSQC spectrum into the ^13^C-, ^15^N-*Xsa*EXLX1 ^1^H-^15^N HSQC spectrum, or vice versa, were unsuccessful. Indeed, while the ^1^H-^15^N HSQC spectra for *Daq*EXLX1 contained roughly the correct number of expected amide chemical shifts, the spectrum for *Xsa*EXLX1 prepared in non-deuterated media (H_2_O) contained ~ 40 fewer. Missing amide ^1^H-^15^N HSQC cross peaks typically identify protein regions undergoing motion or chemical exchange in the ms to ms timescale (intermediate) [40, 41] or heterogeneous protein–protein interfaces [42]. To the best of our knowledge, this is the first time it has been observed that increasing the molecular weight of a protein by the extensive substitution of ^2^H for ^1^H altered the protein’s backbone dynamics. While we continue to explore the physical explanation for the missing amide resonances in the ^1^H-^15^N HSQC spectrum of ^2^H-, ^13^C-, ^15^N-*Xsa*EXLX1, we have assigned ~ 65% of the amide resonances with the extensive assistance of residue-specific ^15^N-labeled amino acid samples (Additional file 1: Fig. S6a). These assignments show that the missing amides are not due to proteolysis and the regions of secondary structure identified through the analysis of assigned chemical shifts (CSI 3.0; http://csi3.wishartlab.com/cgi-bin/index.php) [43] agree with AlphaFold predictions for *Xsa*EXLX1 (Additional file 1: Fig. S6b).

### Binding to polysaccharides

**Fig. 2:**
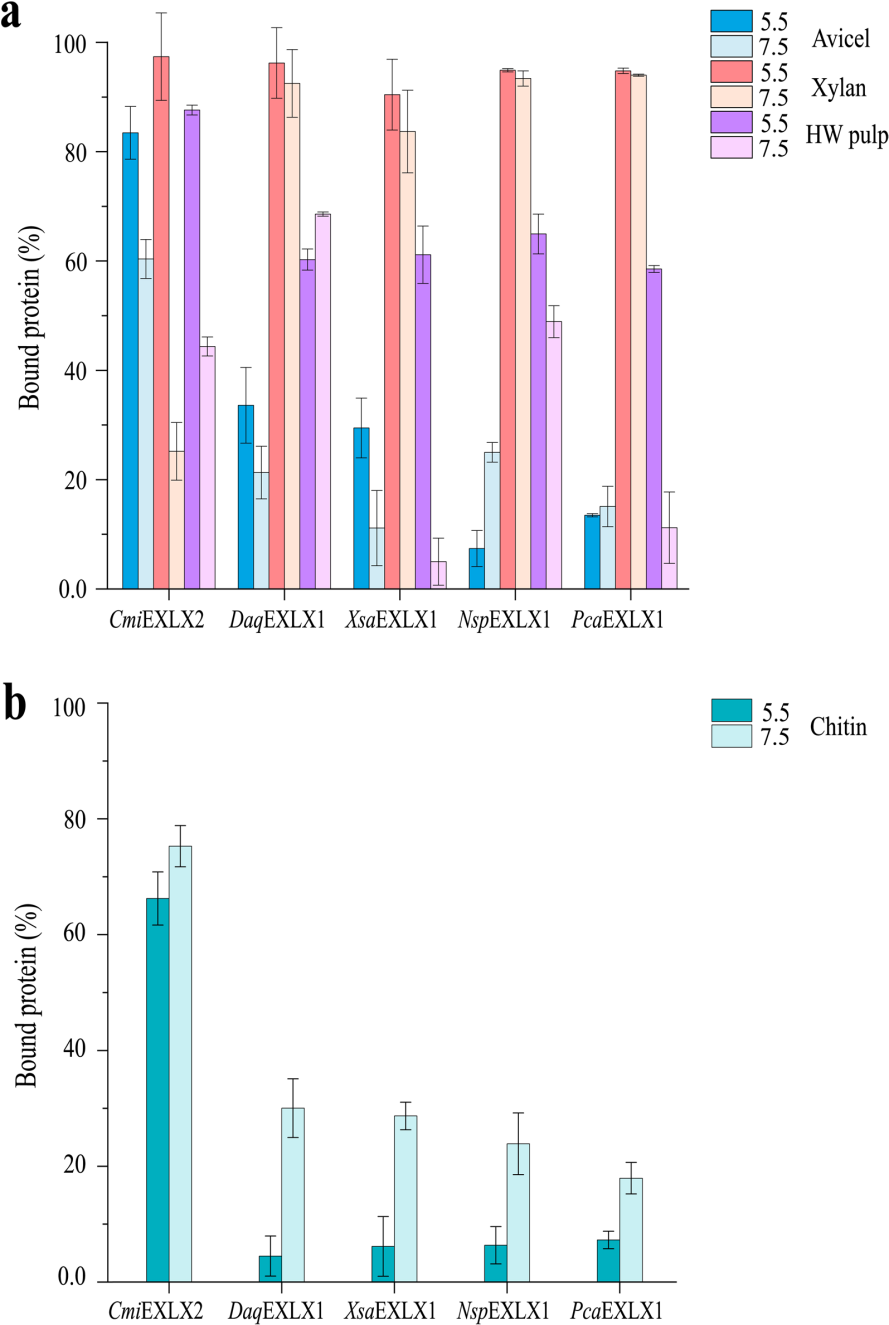
Insoluble Polysaccharide Pull-down (IPP) assay. The binding of recombinantly produced and purified microbial expansins to **a** Avicel (blue), xylan (red), and hardwood pulp (violet) at pH 5.5 (dark) and pH 7.5 (light); and **b** chitin at pH 5.5 (dark) and 7.5 (light). The y-axis indicates the percent of added protein that is bound to the substrate after incubation for 1.5 h. Error bars indicate standard deviations of triplicate experiments

Substrate binding is a prerequisite for function therefore the binding of each microbial expansin to microcrystalline cellulose (Avicel), oat-spelt xylan, hardwood kraft pulp, and chitin was measured at pH 5.5 and pH 7.5 after 1.5 h of incubation. At pH 5.5, each expansin bound best to oat-spelt xylan (Fig. 2a). This was also observed at pH 7.5 except for *Cmi*EXLX2 where binding decreased from ~ 95% to ~ 30% of total protein. Overall, the next best substrate and condition for protein binding was hardwood kraft pulp at pH 5.5. Notably, except for *Cmi*EXLX2, binding to Avicel was comparatively low (between 10 and 30%). Besides plant polysaccharides, the microbial expansins were tested for ability to bind α-chitin. Whereas the microbial expansins bound better to the tested plant polysaccharides at pH 5.5 over pH 7.5, the opposite trend was observed on α-chitin for four of the five microbial expansins (Fig. 2b). Apart from *Cmi*EXLX2 that bound well to chitin at both pH 5.5 and pH 7.5 (65–75% of total protein), the extent of binding to chitin by the other four microbial expansins was low and similar to Avicel. Isothermal titration calorimetry (ITC) measurements also revealed *Cmi*EXLX2 binding to cellopentaose and xylotetraose, whereas binding of oligosaccharides by the other microbial expansins was not detected (Additional file 1: Fig. S7). In addition to the core D1 and D2 domain structure of all microbial expansins tested herein, *Cmi*EXLX2 uniquely contains an N-terminal CBM2 which likely explains the comparatively high binding of this protein to cellopentaose and xylotetraose as well as high molecular weight cellulose, xylan, and chitin [44]. Moreover, the absence of detectable oligosaccharide binding by the other microbial expansins, which comprise only the core D1 and D2 domain structure, is consistent with earlier studies of *Bs*EXLX1 that show binding to such oligosaccharides is entropy driven [45].

### Impact on the hydrolytic and/or oxidative activity of carbohydrate-active enzymes

**Fig. 3:**
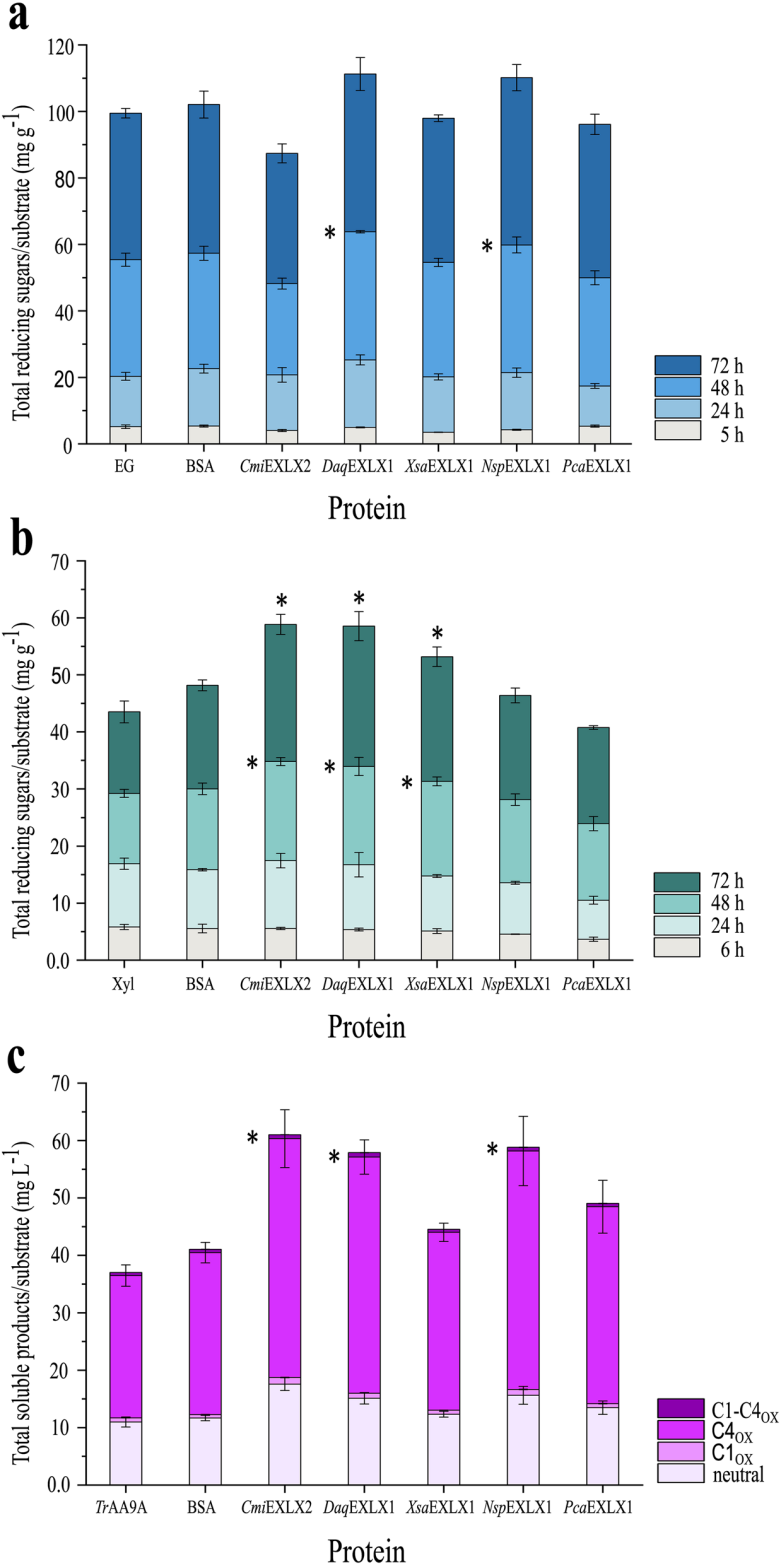
Impact of microbial expansins on hydrolytic and oxidative enzymes used to treat hardwood kraft pulp. The reactions were performed at 40 °C, pH 5.5 up to 72 h. Soluble sugars released were measured in the presence of **a** Endoglucanase (EG) + microbial expansin (EXLX); **b** Xylanase (Xyl) + EXLX; **c** *Tr*AA9A + EXLX. The first, most left column in each panel shows product release by **a** Endoglucanase (EG), **b** Xylanase, and **c** *Tr*AA9A alone. Addition of BSA instead of expansin was used in each case as a reference. Total reducing sugars released in the hydrolysis of pulp by endoglucanase and xylanase after 5/6, 24, 48, and 72 h reaction time were measured using the PAHBAH assay (a and b). In the case of the oxidative *Tr*AA9A enzyme (c), the soluble products were analyzed using UHPLC-IMS-MS at the 72 h time point. Error bars indicate standard deviations for triplicate samples. Asterisks (*) indicate statistically significant (p ≤ 0.05; two-tailed t-test) difference between EXLX and BSA-treated samples

Enhancement of carbohydrate-active enzymes by microbial expansins has been previously reviewed [11, 12]. When surmising impacts of substrate on the potential of microbial expansins to boost lytic enzyme activity, Liu et al. [12] highlighted the apparent benefit of substrates with comparatively high hemicellulose content. Accordingly, the hardwood kraft pulp that contains 22% xylan was used in our studies to investigate the potential of microbial expansins to boost the performance of an endoglucanase, xylanase, and lytic polysaccharide monooxygenase (LPMO). Herein, the *Tr*AA9A LPMO from *T. reesei* was used which catalyzes the oxidative cleavage of glycosidic bonds at both the C4 and C1 carbon positions of cellulose with preference for C4 [46, 47]. Notably, none of the tested microbial expansins appreciably boosted endoglucanase activity (Fig. 3a); however, *Cmi*EXLX2, *Daq*EXLX1, and *Xsa*EXLX1 increased xylanase activity by 24.5%, 26.2%, and 16.8% after 72 h, respectively (Fig. 3b). Moreover, in the presence of *Cmi*EXLX2, *Daq*EXLX1 or *Nsp*EXLX1, *Tr*AA9A released significantly more soluble products (of 1–4 glucose units long) from the hardwood pulp compared to the reference LPMO treatment (in the presence of inert BSA protein) (Fig. 3c, Additional file 1: Table S3). Consistent with earlier studies of *Tr*AA9A on pulp fiber, the majority of soluble products generated by *Tr*AA9A were ketones or gemdiols originating from C4 oxidation, along with small amounts of C1 and double oxidized C1–C4 sugars [46]. In addition, considerable amounts of neutral cello-oligosaccharides were observed, which could arise from the degradation of labile C4 oxidized oligosaccharides, oxidations close to the cellulose chain ends, or indicate depletion of oxygen over time [48]. Whereas the concentration of C4 oxidized sugars released by *Tr*AA9A increased by ~ 31–32% with the addition of microbial expansins, corresponding increases in neutral sugar release varied from ca 22 to 34%. When considering total soluble products liberated by *Tr*AA9A (including neutral, single, and double oxidized), greatest increases in product formation were observed in reactions supplemented with *Cmi*EXLX2, *Nsp*EXLX1, and *Daq*EXLX1, where product yields increased by nearly 40% (Fig. 3c; Additional file 1: Table S3).

### Fibrillation of hardwood kraft pulp

**Fig. 4:**
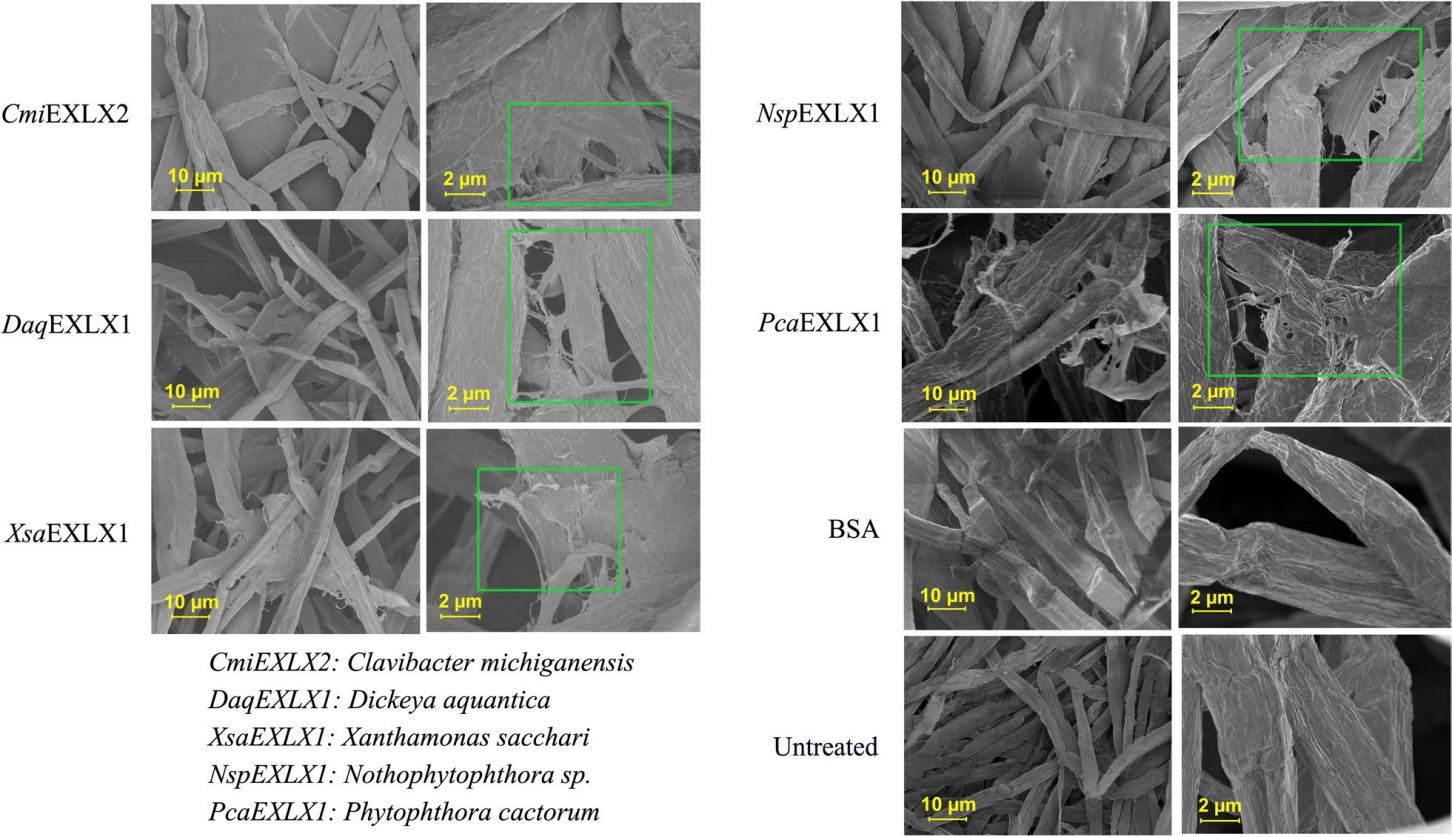
Scanning electron microscopy images of hardwood pulp. Hardwood pulp SEM images after treatment with *Xsa*EXLX1, *Daq*EXLX1, *Cmi*EXLX2, *Nsp*EXLX1, and *Pca*EXLX1 shown at 10 and 2 µM resolution using 500 × and 2500 × magnification, respectively. The reference (without protein) and BSA-treated control samples are shown on the bottom. The major impact of microbial expansin treatments are highlighted in the 2 µm resolution images using phosphoric green frames

**Fig. 5:**
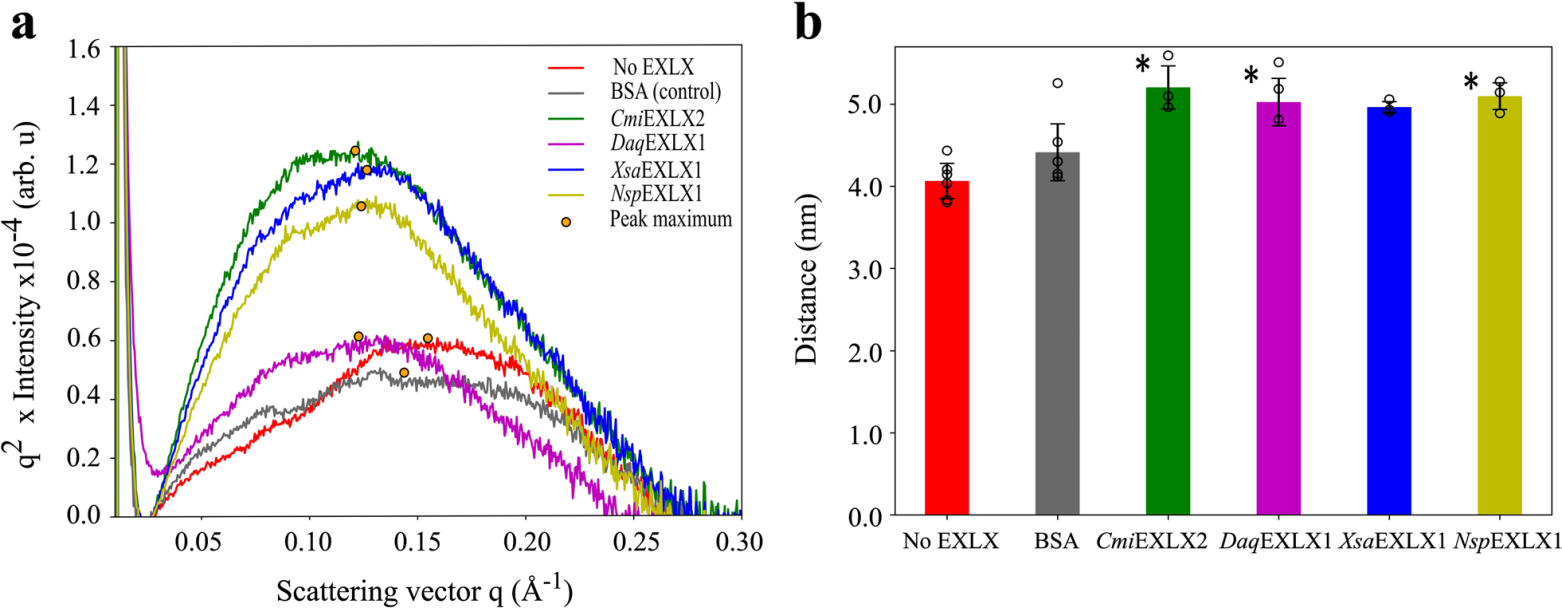
The small-angle X-ray scattering analysis of expansin treated hardwood pulp. **a** SAXS intensities presented in the form of a Kratky plot. **b** The center-to-center distance between fibrils was determined by measuring the location of the peak maximum in the Kratky plot. The two reference samples (i.e., No EXLX and BSA-treated samples) are shown on the left. Error bars indicate standard deviations for parallel samples collected in triplicate. Asterisks (*) indicate statistically significant (p ≤ 0.05; two-tailed t-test) increase in the center–center distance of fibrils in EXLX vs. BSA-treated pulp samples

The potential of the selected microbial expansins to disrupt cellulose fiber and fibril networks was investigated using scanning electron microscopy (SEM), wide-angle X-ray scattering (WAXS), and small-angle X-ray scattering (SAXS). After treatment of kraft hardwood pulp with buffer alone or BSA, SEM images of cellulose fibers appeared uniform with little evidence of fibrillation (Fig. 4). By contrast, SEM images of pulp samples after treatment with the microbial expansins consistently showed evidence of fibrillation (Fig. 4). The parallel WAXS and SAXS analyses were completed for pulp samples treated with *Cmi*EXLX2, *Daq*EXLX1, *Xsa*EXLX1, and *Nsp*EXLX1 given the beneficial impact of those microbial expansins on xylanase or *Tr*AA9A activity. WAXS analysis of the pulp samples did not distinguish the reference pulp from those treated with the microbial expansins, where neither the *d*-spacing (from peak location) nor the crystal size (from peak width) in any lattice direction were notably altered. Changes in the crystallinity index were also minimal, with more variation between individual samples than between the different treatments. By contrast, SAXS analyses did distinguish the reference pulp samples from those treated with the microbial expansins. The scattering in the SAXS region around values of the scattering vector *q* = 0.1 Å^−1^ and above (structure size 6 nm and below) arise mainly from the lateral size of cellulose microfibrils (diameter 3–5 nm) and their mutual packing [49]. The Kratky plot (q^2 * I vs. q (vector)) for three of the four microbial expansin treated samples shows a clear peak shift to the left, indicating either an increase in fibril diameter or interfibrillar spacing (Fig. 5a, b). Because the WAXS profiles and especially the crystal size did not change, the shift in SAXS profiles can be interpreted as an increase in the interfibrillar spacing within 20–25% for all cellulose samples treated with *Cmi*EXLX2, *Daq*EXLX1, or *Nsp*EXLX1 (Additional file 1: Fig. S8).

## Discussion

The presence of protein domains beyond the D1 and D2 expansin core is expected to impact the biological function and applied potential of these proteins. For example, fungal swollenins contain an N-terminal CBM1 that reportedly improve protein binding and amorphogenesis of cellulosic materials [22, 31]. Bacterial expansin-related proteins have been identified that harbor N-terminal or C-terminal CBM2, CBM3, CBM6, or GH5 modules which could play similar roles in protein targeting and action [35]. Herein, the binding profiles of *Cmi*EXLX2 that uniquely comprises an N-terminal CBM2 were differentiated from the other characterized microbial expansins by its ability to bind both soluble cello- and xylooligosaccharides and comparatively high binding to microcrystalline cellulose (Avicel) and chitin.

**Fig. 6:**
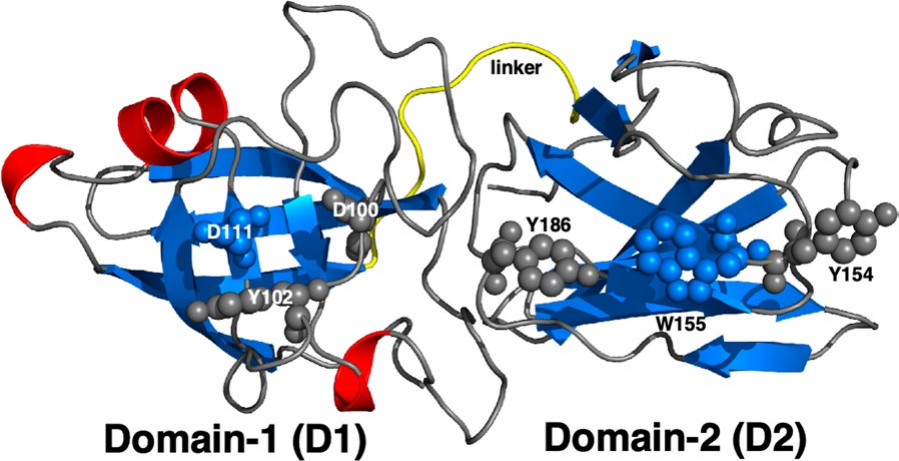
Cartoon representation of the AlphaFold predicted for *Xsa*EXLX1 structure (www.alphafold.ebi.ac.uk). A six-residue linker region, highlighted in yellow, joins two tightly packed domains, D1 and D2, observed in the solved crystal structures of related plant and microbial expansins. The β-strands and α-helices are colored in blue and red, respectively. The N-terminal D1 domain adopts a double-Y barrel fold (DPBB) and the C-terminal D2 domain adopts a β-sandwich with an Ig-like fold. Three conserved residues in the D1 domain, D100, Y102, and D111 (side chains highlighted as spheres), contribute to expansin function via an unknown mechanism. Three conserved and linearly arranged aromatic residues in the D2 domain, Y154, W155, and Y186 (side chains highlighted as spheres) are expected to promote binding to cellulosic structures. Together these two regions on both domains form one continuous surface on the same face of *Xsa*EXLX1

In the absence of additional CBMs, substrate binding by microbial expansins is largely mediated through aromatic amino acids in the D2 domain. Extending the corresponding face of the D2 domain is the shallow groove of the D1 domain that is predicted to form a polysaccharide binding surface (PBS) [13, 50]. It has been hypothesized that electrostatic surfaces on the face opposite the PBS play a role in substrate differentiation among microbial expansins and affect expansin activity on cellulose [10, 51]. This was supported by an earlier electrostatic analysis of I-TASSER-based models of bacterial expansins which delineated Gram-positive proteins with an acidic surface opposite the PBS face and Gram-negative proteins with a basic surface opposite the PBS face [52]. A surface charge analysis of the microbial expansins characterized herein at pH 7 was conducted using structures modeled with AlphaFold, a protein modeling program more accurate than I-TASSER [53, 54]. As illustrated (Fig. 6; Additional file 1: Fig. S9), all five microbial expansins contained a large negatively charged surface on the PBS face centered around D82 in the D1 domain (top structures). On the opposite face (bottom structures) the feature consistent with all five structures was a negatively charged surface in the D1 domain; the surface of the D2 domain was neutral (white) or speckled with small negatively or positively charged regions. Since this analysis included both Gram-negative and Gram-positive sequences, and pH-dependent substrate binding profiles of the corresponding expansins differed despite having similar predicted surface charge distribution, the calculated surface change (at pH 7.0) on the face opposite the PBS does not appear sufficient to predict substrate preference. The sample size was small, however, and to better explore the impact of surface charge on expansin activity it will be important to measure the impact of pH on both the surface charge of the protein and the targeted substrate.

In the absence of lytic activity, it is hypothesized that cell wall loosening by the microbial expansin is achieved through disrupting non-covalent bonds between cellulose microfibrils in the plant cell wall [6]. The unique observation that perdeuteration of one of the microbial expansins in this study, *Xsa*EXLX1, resulted in the disappearance of approximately 40 amide resonances in its ^1^H-^15^N HSQC spectrum suggests that at least part of the protein may be tethering at the edge of motion or chemical exchange in the ms to ms timescale (intermediate). Because such intermediate timescale motion is often associated with regions involved in catalysis and ligand binding [50], perhaps such dynamics plays a vital role in disrupting non-covalent bonds between cellulose microfibrils.

Elucidating the mode of expansin action will require complementary biophysical approaches. For example, robust biomechanical weakening assays have been established to study the action of plant and microbial expansins, and benefit from custom-built extensometers that permit measurements at small-scale and low protein quantities [16, 51]. Additional biophysical methods to investigate the impacts of microbial expansins on cellulose fiber include light microscopy [22, 55], atomic force microscopy [27], scanning electron microscopy (SEM) [25, 56], X-ray diffraction [25], and nitrogen adsorption [26]. These analyses have revealed varying impacts of microbial expansins on cellulosic substrates that depend on the cellulose source and pretreatment, as well as the use of native or recombinant protein preparations [27]. Even though it is still difficult to predict expansin performance based on protein sequence, convincing evidence points to the widespread potential of microbial expansins to disrupt cellulosic networks. This is underscored by the SEM analyses performed herein that showed fibrillation of hardwood pulp after treatment with microbial expansins from diverse phylogenetic and taxonomic origin. Moreover, the combined WAXS and SAXS analyses included in the current study permit quantitative assessment of expansin action on cellulosic materials that distinguish change in the diameter and packing distance of the microfibrils. As in earlier studies that used WAXS to compare Avicel samples before and after treatment with *Tr*SWO1 [27] or SWO2 from *Trichoderma pseudokoningii* S38 [28], a change in crystallinity of the pulp samples after treatment with microbial expansins was not observed. Instead, parallel SAXS measurements clearly showed an increase in interfibrillar distance between neighboring cellulose fibrils in the pulp samples after expansin treatment. These results indicate that for at least some microbial expansins, their action increases the available surface area of cellulosic substrates by disrupting interfibrillar associations rather than necessarily reducing the crystallinity of the individual cellulose fibrils. The potential of the microbial expansins characterized herein to expose cellulose fibril surfaces while not reducing fiber crystallinity may explain the measured benefits that certain microbial expansins had on xylanase and LPMO activity on degrading hardwood kraft pulp. For example, given the expected localization of hemicelluloses on cellulose fibril surfaces, greater exposure of the surface would increase xylanase accessibility to the targeted xylan substrate. Similarly, fibrillation and increased accessibility to fibril surfaces without apparent reduction in cellulose crystallinity could improve oxidative action of LPMOs on celluloses.

## Conclusion

The direct comparison of microbial expansins from diverse taxonomic origin and modular structure reported herein points to widespread potential of microbial expansins to alter cellulose networks. The application of SAXS together with WAXS to quantify such changes can help uncover impacts of protein sequence and substrate composition on the mode of expansin action. Predicting protein performance and substrate preference from protein sequence, however, will likely require additional attention be directed to quantifying impacts of treatment conditions (e.g., pH) on both protein and substrate properties including surface charge. Moreover, evaluating microbial expansins on substrates besides plant materials (e.g., bacterial and fungal cell wall components) is needed to assess the substrate selectivity and biological function of this protein family. Ultimately, it is anticipated that a deeper understanding of the microbial expansin family can lead to novel process technologies to create high-yield materials from renewable bioresources.

## Material and methods

### Substrates and enzymes

Oven-dried (60 °C) hardwood kraft pulp (98% dry matter content) was kindly provided by UPM-Kymmene Oyj (Lappeenranta, Finland). Cellopentaose and xylotetraose were purchased from Megazyme (Bray, Ireland). A commercial xylanase (Ecopulp TX-800, AB Enzymes) and cellulase (*endo*-1,4-β-D-glucanase, GH12 from *Aspergillus niger*, 600 U mL^−1^, CAS No. 9012–54-8, Megazyme) were used in the enzyme studies, along with the *T. reesei* family 9 LPMO (*Tr*AA9A) that was produced and purified as previously described [46].

### Microbial expansin sequence analysis and selection

In total, 4178 putative expansin-related proteins were retrieved by a BlastP search against the non-redundant protein database via the NCBI server (https://blast.ncbi.nlm.nih.gov/Blast.cgi) using *Bs*EXLX1 (PDB entry: 4FER) as a query sequence. The retrieved sequences were filtered by excluding plant expansins and fungal expansin-related proteins that displayed < 30% sequence identity and < 80% coverage to the query. Sequences comprising 195–600 amino acids were aligned and curated using MAFFT [57] with default parameters. The alignment was used to construct a phylogenetic tree using NGphylogeny [58] and rendered by iTOL [59].

### Microbial expansin cloning and protein production

Sixteen bacterial and five eukaryotic gene sequences were selected and codon optimized for expression in *E. coli* and *P. pastoris,* respectively (GenScript Biotech, the Netherlands, B.V.). In all cases, the native signal sequence for secretion was removed. The bacterial genes were cloned at the NcoI/XhoI restriction sites and expressed using the pelB signal sequence of the pET-22b( +) expression vector with a C-terminal (His)_6_ tag (GenScript Biotech, the Netherlands, B.V.). The resulting plasmids were propagated in *E. coli* TOP10 and transformed into *E. coli* strain BL21 (DE3) for protein production. The eukaryotic genes were cloned at the XhoI/XbaI restriction sites of the *p*PICZαA vector and expressed under the AOX1 promoter using the alpha factor signal sequence and C-terminal (His)_6_ tag (Invitrogen, Carlsbad, CA, USA); plasmids were linearized with PmeI and transformed into competent *P. pastoris* X-33 and KM71H cells by electroporation using the Easy Select Expression System protocol (Invitrogen).

For bacterial protein production, 2 mL pre-cultures of each *E. coli* transformant were grown for up to 5 h at 37 ºC in Luria–Bertani (LB) broth supplemented with 100 µg mL^−1^ ampicillin and then transferred to 400–600 mL LB in 2–3 L shake flasks and grown at 30 ºC until an *OD*_600_ of 0.4–0.6 was reached. The culture temperature was then reduced to ~ 18 °C before adding 0.5–1.0 mM isopropyl β-d-1-thiogalactopyranoside (IPTG). Following induction for 18 h, the cultures were harvested by centrifugation (4000 rpm, 20 min, 4 °C) and the resulting cell pellets suspended in 2 mL g^−1^ (cell weight) of Tris buffer (pH 7.8) containing 150 mM NaCl and 10 mM imidazole before sonication in an ice bath using a Qsonica Q500 sonicator with a 2 mm probe. The sonicator was programmed to operate at 25 kHz amplitude for 7 min in pulsed mode (2 s on and 5 s off). Following sonication, the material was centrifuged (8000 × g, 30 min, 4  °C) and the clarified supernatants passed through a 0.45 µm filter prior to purification using column chromatography.

For eukaryotic protein production, pre-cultures were grown for 6 h at 30 °C in 5 mL YPD (Yeast Extract–Peptone–Dextrose) medium and then transferred to 500 mL BMGY (Buffered Glycerol-complex Medium, Invitrogen) in 2 L non-baffled shaker flasks and grown at 30 °C until reaching an *OD*_600_ of 4–6. Cultivations were centrifuged (4000 × g, 10 min, 4  °C), the cells pellets were suspended in BMMY (Buffered Methanol-complex Medium) medium (one fifth volume of BMMY, 0.002% biotin), and gene expression was induced at 20 °C for up to 75 h by the addition of 3% methanol by 24 h intervals. Following induction, cultures were harvested by centrifugation (5000 × *g*, 10 min, 4 °C) and clarified supernatants were adjusted to pH 7.8 using 2 M NaOH before filtration through 0.45 µm filters (Millipore Express® PLUS, PES Membrane).

Recombinantly produced protein were purified using a 5 mL His Trap HP column (Cytiva, Uppsala, Sweden) connected to an Äkta purifier 100 FPLC system (GE Healthcare) and equilibrated with buffer A (50 mM Tris–HCl, pH 7.8, 150 mM NaCl, 10 mM imidazole). The samples were loaded on the column at 2.5 mL min^−1^; unbound proteins were removed using 15 column volumes (CVs) of buffer A, and bound proteins were eluted with 0–40% buffer B (50 mM Tris–HCl, pH 7.8, 150 mM NaCl, 500 mM imidazole) over 10 CVs. Protein purity was verified by SDS-PAGE analysis and selected fractions containing the target protein were pooled, concentrated, and exchanged to 50 mM sodium acetate buffer (pH 5.5) using 10 kDa Vivaspin 20 ultrafiltration units (Sartorius, Göttingen, Germany). Protein concentration was determined by measuring *A*_280_ using a Nanodrop ND-2000 (Thermo Fischer Scientific). The theoretical molar extinction coefficient of each protein was calculated using the Protparam tool on the EXPASY server (web.expasy.org/protparam/) (Additional file 1: Table S1).

Nitrogen-15 labeled *Daq*EXLX1 was prepared using conventional autoinduction methods [60]. Triple-labeled (^2^H- ^15^N-, ^13^C-) *Xsa*EXLX1 was obtained by growing the transformed cells (37 °C) in 750 mL of minimal medium (Miller) in a baffled 2L flask containing 98% ^2^H_2_O (v), ^15^NH_4_Cl (1 mg mL^−1^) and D-[^13^C_6_]glucose (2.0 mg mL^−1^), NaCl (50 mg mL^−1^), MgSO_4_ (120 mg mL^−1^), CaCl_2_ (11 mg mL^−1^), and the antibiotics ampicillin (150 mg mL^−1^). This was accomplished by adding an ~ 1 mL frozen glycerol stock (BL21(DE3); OD_600_ ~ 0.8) to 20 mL of the above minimal media solution and adding this directly to the 750 mL minimal media solution upon reaching an OD_600_ of ~ 0.7. When the 750 mL cell culture reached an OD_600_ reading of ~ 0.8, it was transferred to a 25 °C incubator and gene expression induced with IPTG (0.026 mg mL^−1^). Cells were harvested by mild centrifugation following overnight incubation and then frozen at − 80 °C. Double-labeled (^15^N-, ^13^C-) *Xsa*EXLX1 was prepared identically except for the use of H_2_O instead of D_2_O in the media. Due to poor magnetization transfer in the backbone assignment NMR experiments even with perdeuterated samples, eight residue-specific, ^15^N-labeled amino acid (A, K, R, V, L, A, H, Y) samples were also prepared by growing the cells in highly supplemented “Redfield-medium” [61]. For the residue-specific samples, the cells were harvested ~ 1 h after IPTG induction. Following the thawing of frozen pellets, all the labeled samples were purified with a conventional two-step protocol involving metal chelate affinity chromatography on a 20 mL Ni-Agarose 6 FastFlow column (GE Healthcare, Piscataway, NJ) followed by gel-filtration chromatography on a Superdex75 HiLoad 26/60 column (GE Healthcare, Piscataway, NJ). In addition to removing minor impurities, the latter step exchanged the microbial expansins into the buffer used for the NMR studies: 100 mM NaCl, 20 mM Tris, 1.0 mM dithiothreitol, pH 7.0.

### Nano differential scanning fluorimetry (NanoDSF)

The thermal unfolding of target proteins was monitored using a Prometheus NT.48 instrument equipped with capillary tubes. Each measurement consumed 10 μL of protein prepared in 50 mM sodium acetate (pH 5.5); protein concentrations were adjusted to 2 mg mL^−1^ or 1.0 mg mL^−1^ depending on the number of tyrosine and tryptophan residues, respectively. The sample temperature was increased from 20 °C to 95 °C at 1 °C min^−1^ and the fluorescence intensity at emission wavelengths between 330 and 350 nm recorded.

### Circular dichroism (CD) spectroscopy

The CD spectra of purified target proteins were obtained using a Chirascan™ CD spectrophotometer (Applied Photophysics Ltd) in a quartz cuvette with 1 mm path length. All protein concentrations were adjusted to 0.1–0.15 mg mL^−1^ in 25 mM sodium acetate buffer (pH 5.5). A wavelength spectrum from 180 to 280 nm was recorded in triplicate at 25 °C. To measure thermal denaturation, CD spectra between 190 and 260 nm were collected at 2 °C intervals (1 °C min^−1^ ± 0.2 °C tolerance) using a QUANTUM temperature controller. Data analysis was conducted using Pro-Data viewer (Applied Photophysics Ltd.), CDNN (http://bioinformatik.biochemtech.uni-halle.de/cdnn), and Global3 (Applied Photophysics Ltd.). A baseline spectrum for 25 mM sodium acetate buffer, pH 5.5, was also collected.

### Nuclear magnetic resonance (NMR) spectroscopy

The NMR data used for the chemical shift assignments were collected at 30 °C on a triple-labeled (^2^H-, ^13^C-, ^15^N-) sample of *Xsa*EXLX1 (~ 0.5 mM) using Agilent Inova-600 spectrometers equipped with an HCN-cryoprobe and pulse field gradients. Chemical shift assignments were made primarily through the analysis of HNCA, HNCOCA, HNCACB-(^13^Cb-optimized), CBCA(CO)NH, HNCO, HNCACO, and ^15^N-edited NOESY-HSQC three-dimensional experiments (often optimized for fully deuterated proteins) using Agilent Biopack pulse programs. While the ^1^H-^15^N HSQC spectra for the residue-specific, ^15^N-labeled samples corresponded to a simplified ^1^H-^15^N HSQC spectrum of non-deuterated *Xsa*EXLX1, unambiguous assignments for deuterated *Xsa*EXLX1 could be made taking all the available NMR data into account. Unsuccessful efforts to convert the ^2^H-, ^13^C-, ^15^N-*Xsa*EXLX1 ^1^H- ^15^N HSQC spectrum into the ^13^C-, ^15^N-*Xsa*EXLX1 ^1^H- ^15^N HSQC spectrum, or vice versa, included changing the temperature, changing the protein concentration, adding reducing agents (TCEP or DTT), and adding a chelating agent (EDTA). Felix 2007 (MSI, San Diego, CA) and POKY were used to process and analyze all the NMR data.

### Isothermal titration calorimetry (ITC)

ITC was conducted using a MicroCal iTC-200 microcalorimeter (Malvern). Three hundred µL of the target protein (50 µM, 20 mM sodium acetate buffer, pH 5.5) were loaded into the sample cell and 60 µL ligand (0.5 M cellopentaose or xylotetraose, 20 mM sodium acetate buffer, pH 5.5) were loaded into the injection syringe. Experiments were performed at 25 °C and all cell-syringe preparations were followed according to the manufacturer’s instructions (MicroCal iTC-200, Malvern). Binding titrations were initiated by a 0.4 µL injection followed by 3.6 µL injections at 180 s intervals. Data analyses were performed using the ORIGIN software (OriginLab) with the first injection excluded as per the manufacturer’s instruction.

### Insoluble polysaccharide pull-down (IPP) assay

The IPP substrates Avicel (PH-101), oat-spelt xylan, and chitin were washed twice with deionized water, 70% ethanol, and then washed twice with MilliQ water. The assays were performed in 1.5 mL Eppendorf tubes using 250 µL 1% (w/v) ligand plus protein (0.2 mg mL^−1^) prepared in either 50 mM sodium acetate pH 5.5 or 50 mM HEPES buffer pH 7.5. All assays were incubated at 25 °C for 90 min on a ThermoMixer (Eppendorf AG, Hamburg, Germany) set to 1100 rpm orbital shaking and performed in triplicate. Following centrifugation before and after the addition of ligand, the protein concentrations were measured using the Bradford (Hercules) or BCA assay (Pierce™ BCA Protein Kit, ThermoFisher Scientific, USA). The percentage of protein bound to the ligand was calculated as (1– (final protein concentration/initial protein concentration) × 100).

Boosting impact of microbial expansins on carbohydrate-active enzymes.

Reactions (final volume 250 µL) were performed in 1.5 mL Eppendorf tubes containing 2.5 mg (1% w/v) hardwood pulp in 50 mM sodium acetate buffer, pH 5.5. All reactions were incubated at 40 °C with shaking at 950 rpm using a ThermoMixer (Eppendorf, Hamburg, Germany). Samples (25 µL) were collected for analyses at 6, 24, 48, and 72 h. Reactions with hydrolytic enzymes were supplemented with 9 mg g^−1^ of microbial expansin or BSA per gram of pulp, and 0.5 mg g^−1^ of xylanase (TX-800) or cellulase (EG) per gram of pulp. Reactions with the *Tr*AA9A LPMO were supplemented with 12 mg g^−1^ pulp of microbial expansin or BSA, and 0.5 mg g^−1^ pulp of *Tr*AA9A. In all cases, the enzyme loading ensured an ability to measure enhanced enzyme performance over the 72 h incubation. For the xylanase and cellulase treatments, total solubilized reducing sugars were measured using the 4-hydroxybenzoic acid hydrazide (PAHBAH) assay [62]. Briefly, 10 µL of the sample were mixed with 200 µL of the PAHBAH reagent, heated to 70°C for 30 min, and cooled to 4 °C, and the absorption at *A*_405nm_ was measured (BioTek PowerWave model)**.** The profile of sugars released by the hydrolytic enzymes was measured using a standard curve prepared from glucose (0.01–0.3 mg mL^−1^). For the *Tr*AA9A treatments, the separation and detection of soluble oligosaccharides were carried out using liquid chromatography on a Acquity UPLC system (Waters, Milford, MA, USA) with a HYPERCARB column (Thermo Scientific) combined to a Synapt G2-S mass spectrometry (Waters, Milford, MA, USA) in ESI-positive ion mode and traveling wave ion mobility (TWIM), as described previously^45^. The relative quantities of the different sugars were determined using calibration curves made from non-oxidized cello-oligosaccharides (0.05 to 100 µg mL^−1^ with a 1–4 degree of polymerization (DP), in water).

### Scanning electron microscopy (SEM)

A 250 µL solution of 1% w/v hardwood pulp and 0.2 mg mL^−1^ protein in 50 mM sodium acetate buffer, pH 5.5, were incubated in 1.5 mL Eppendorf tubes at 40 °C for 72 h on a ThermoMixer set to 1100 rpm orbital shaking. Control experiments without protein were treated similarly. Pulp samples were washed twice with MilliQ water, dried at 45 °C, placed on sample stubs with double sided carbon tape, and sputtered for 15 min (20 mA current) using an EM ACE200 vacuum coater (Leica, Germany) to obtain ~ 3 nm of Au/Pd coating prior to SEM analysis. Images were collected using a secondary electron detector on a Sigma VP (Zeiss) SEM with an accelerating voltage of 1.2–1.5 keV.

Wide- and small-angle X-ray scattering (WAXS and SAXS).

Hardwood pulp samples were treated and dried as described for the SEM analyses. Each sample was then packed inside a 1-mm-thick metal washer and covered on both sides with Kapton tape. Upon placing the sample in the washer, random orientation of the fibers was targeted. Data were collected on a Xenocs Xeuss 3.0 C SAXS/WAXS device using a GeniX 3D Cu X-ray source (wavelength λ = 1.542 Å) and an EIGER2 R 1M detector. An empty sample holder (washer and Kapton tape only) was used to measure the background.

Both small-angle (SAXS) and wide-angle X-ray scattering (WAXS) data were collected for each sample. The WAXS data were collected at a sample-to-detector distance of 55 mm, covering a range from ~ 0.1 to 3.0 Å^−1^ for the scattering vector *q*, with the image acquisition taking 2400 s (2700 s for *Daq*EXLX1 and its background). The SAXS data were collected at a sample-to-detector distance of 600 mm, covering a *q*-range from ~ 0.01 to 0.3 Å^−1^, with the image acquisition taking 1000 s (600 s for *Daq*EXLX1 and its background). Relative to the WAXS measurements, the SAXS measurements employed a beam with 4.5 times higher X-ray intensity to compensate for shorter acquisition times. All scattering intensities shown in the figures are averages of at least three parallel samples.

The 2D scattering images were azimuthally integrated to obtain 1D intensity vs. *q* profiles for both the SAXS and WAXS data. The 1D WAXS intensities corresponding to the four most prominent cellulose I_β_ peaks (1–10, 110, 102, 200) and a broad amorphous contribution below them were fitted with Gaussian profiles. According to Bragg’s law, the peak location, *q*_*max*_, yields the *d*-spacing of the lattice planes in the cellulose crystallites:

1 $$d = { 2}\pi /q_{max}$$

According to the Scherrer equation the full width at half maximum of each peak $\Delta q$ yields the crystal size (*L*):

2 $$L=\frac{2\uppi }{ \Delta q}.$$

The crystallinity index was determined by comparing the area of the crystalline peaks to the total area under the 1D WAXS intensity curve. The 1D SAXS intensities were analyzed by first subtracting a *q*^−*α*^ power law (*α* = 4 for dry samples) fitted at low *q* and a constant background fitted at high *q.* The remaining intensity was plotted as a Kratky plot (*q*^*2*^*I* vs. *q*) which showed a clear peak for all samples. The peak maximum was determined by fitting a Gaussian function to the Kratky plot. The location of the peak maximum was converted to an estimate of the average center-to-center distance between the microfibrils using Bragg’s law (Eq. 1).

### Supplementary Information

**Additional file 1: Table S1.** Microbial expansin proteins selected for recombinant expression. **Table S2.** Summary of the target recombinant microbial expansin protein yields. **Table S3.** Sugars analysis by LC-MS. **Figure S1.** Sequence similarity/identity among our targeted microbial expansins. **Fig. S2 **Differential Scanning Fluorimetry (DSF) experiments. **Figure S3.** Circular dichroism (CD) analysis. **Figure S4.** NMR analysis of two bacterial expansins.** Figure S5.** Perdeuteration of *Xsa*EXLX1 increases intermediate (ms to ms) motion**. Figure S6.** Current chemical shift assignment for *Xsa*EXLX1 corroborates predicted AlphaFold structure.** Figure S7.** Isothermal titration calorimetry (ITC). **Figure S8.** X-ray scattering analysis.** Figure S9.** Predicted electrostatic surface potentials of the targeted microbial expansins.

## Data Availability

All generated or analyzed data during this study are included in this published article and its Additional files.

## Abbreviations

CBM: Carbohydrate-binding module
GH: Glycoside hydrolase
EXLX: Microbial expansin-like protein
LPMO: Lytic polysaccharide monooxygenase
WAXS: Wide-angle X-ray scattering
SAXS: Small-angle X-ray scattering
SEM: Scanning electron microscopy
