# Supplementary material for: Insights into the action of phylogenetically diverse microbial expansins on the structure of cellulose microfibrils
Source: Biotechnol Biofuels Bioprod. 2024 Apr 23;17:56. doi: 10.1186/s13068-024-02500-w (PMC11040781; doi:10.1186/s13068-024-02500-w)
Supplement: Supplementary file 1 — Additional file 1: Table S1. Microbial expansin proteins selected for recombinant expression. Table S2. Summary of the target recombinant microbial expansin protein yields. Table S3. Sugars analysis by LC-MS. Figure S1. Sequence similarity/identity among our targeted microbial expansins. Fig. S2 Differential Scanning Fluorimetry (DSF) experiments. Figure S3. Circular dichroism (CD) analysis. Figure S4. NMR analysis of two bacterial expansins. Figure S5. Perdeuteration of XsaEXLX1 increases intermediate (ms to ms) motion. Figure S6. Current chemical shift assignment for XsaEXLX1 corroborates predicted AlphaFold structure. Figure S7. Isothermal titration calorimetry (ITC). Figure S8. X-ray scattering analysis. Figure S9. Predicted electrostatic surface potentials of the targeted microbial expansins. [file 13068_2024_2500_MOESM1_ESM.docx]

**Additional file 1**

Insights into the action of phylogenetically diverse microbial expansins on the structure of cellulose microfibrils

Majid Haddad Momeni^1^*, Aleksi Zitting^1^, Vilma Jäämuru^1^, Rosaliina Turunen^1^, Paavo Penttilä^1^, Garry W. Buchko^2,2+^, Salla Hiltunen^3^, Natalia Maiorova^4^, Anu Koivula^4^, Janak Sapkota^3^ Kaisa Marjamaa^4^, Emma R. Master ^1,5^*

^1^ Department of Bioproducts and Biosystems, Aalto University, Kemistintie 1, Espoo 02150, Finland

^2^ Earth and Biological Sciences Directorate, Pacific Northwest National Laboratory, Richland, WA 99354, United States of America

^2+^ School of Molecular Biosciences, Washington State University, Pullman, WA 99164, United States of America

^3^ NE Research Center, UPM Pulp Research and Innovations, 53200 Lappeenranta Finland

*^4^* VTT Technical Research Centre of Finland Ltd, P.O. Box 1000, 02044-VTT, Espoo, Finland

^5^ Department of Chemical Engineering and Applied Chemistry, University of Toronto, 200 College Street, Toronto, ON, M5S 3E5, Canada

* Correspondence:

**Emma R. Master**

Email: [emma.master@aalto.fi](mailto:emma.master@aalto.fi), emma.master@utoronto.ca

Phone number: +1416-946-7861

**Majid Haddad Momeni**

Email: [majid.haddad@aak.com](mailto:majid.haddad@aak.com)

Phone number: +4676-854-4600

| **Table S1.** **Microbial** **expansin proteins selected for recombinant expression**. | | | | | | | |  |
| --- | --- | --- | --- | --- | --- | --- | --- | --- |
| Organism | GenePept accession no. | Name | Full  length | length^2^ | pI^2^ | Size^2^  (kDa) | modularity | |
| *Xanthamonas sacchari* | AJC47165.1 | *Xsa*EXLX1^1^ | 237 | 217 | 6.82 | 23.40 | EXLX | |
| *Xanthamonas vasicola* | WP_010376099.1 | *Xva*EXLX1 | 589 | 577 | 7.88 | 60.48 | GH5-EXLX^1^ | |
| *Dickeya aquatica* | SLM63089.1 | *Daq*EXLX1 | 231 | 217 | 8.42 | 23.63 | EXLX | |
| *Dickeya solani* | AUC40652.1 | *Dso*EXLX1 | 234 | 216 | 6.16 | 23.65 | EXLX | |
| *Clavibacter michiganensis* | WP_015488949.1 | *Cmi*EXLX2 | 360 | 341 | 5.78 | 35.57 | CBM2-EXLX^1^ | |
| *Streptomyces sp.* | WP_234442800.1 | *Ssp*EXLX1 | 241 | 211 | 7.84 | 23.04 | EXLX | |
| *Nothophytophthora sp.* | RLN20739.1 | *Nsp*EXLX1 | 226 | 213 | 8.50 | 23 | EXLX | |
| *Phytophthora cactorum* | KAG2772387.1 | *Pca*EXLX1 | 223 | 212 | 8.30 | 23.1 | EXLX | |
| *Xanthomonas arboricola* | WP_102255603.1 | *Xar*EXLX1 | 590 | 578 | 6.74 | 60.5 | GH5-EXLX | |
| *Ralstonia solanacearum* | BCL92919.1 | *Rso*EXLX1 | 256 | 230 | 9.24 | 24.78 | EXLX | |
| *Dactylosporangium sp.* | NNJ61190.1 | *Dsp*EXLX1 | 408 | 360 | 4.54 | 36.96 | EXLX-CBM2 | |
| *Dactylosporangium sp.* | MBT8226356.1 | *Dsp*EXLX2 | 303 | 259 | 8.64 | 26.71 | EXLX | |
| *Micromonospora orduensis* | WP_139583295.1 | *Mor*EXLX1 | 302 | 257 | 9.24 | 26.48 | EXLX | |
| *Aquimarina algicola* | WP_140592451.1 | *Aal*EXLX1 | 472 | 461 | 5.92 | 50.96 | EXLX-CBM6^1^ | |
| *Vibrio mangrovi* | WP_087481129.1 | *Vma*EXLX1 | 335 | 324 | 4.39 | 34.84 | EXLX-CBM2 | |
| *Myxococcus xanthus* | WP_216609154.1 | *Mxa*EXLX1 | 231 | 219 | 5.67 | 23.84 | EXLX | |
| *Streptomyces sp.* | WP_234442800.1 | *Ssp*EXLX1 | 241 | 211 | 7.84 | 23.04 | EXLX | |
| *Streptomyces sp.* | WP_230216562.1 | *Ssp*EXLX2 | 297 | 278 | 9.72 | 29.05 | EXLX | |
| *Mitsuaria sp.* | WP_175538275.1 | *Msp*EXLX1 | 339 | 319 | 6.04 | 33.35 | EXLX-CBM2 | |
| *Phytophthora kernoviae* | RLN55428.1 | *Pke*EXLX1 | 237 | 227 | 4.54 | 24.15 | EXLX | |
| *Phytophthora infestans* | KAF4132858.1 | *Pin*EXLX1 | 293 | 283 | 4.43 | 30.39 | EXLX | |
| *Dictyostelium purpureum* | XP_003291366.1 | *Dpu*EXLX1 | 223 | 211 | 6.04 | 23.36 | EXLX | |
| ^1^ EXLX = Microbial expansin with “D1” and “D2” domains. Some microbial expansins contain an additional domain: GH5 = glycoside hydrolase family 45; CBM2 = carbohydrate binding module family 2; CMB6 = carbohydrate binding module family 6.  ^2^ All recombinant proteins were produced without their native signal peptides as C-terminal fusions to an “AAAHHHHHH” purification tag. The length, pI number, and size are calculated based on the processed mature protein using the ProtParam online server (https://web.expasy.org/cgi-bin/protparam/protparam). | | | | | | | |  |

| **Table S2. Summary of the target recombinant microbial expansin protein yields.** | | | | | |
| --- | --- | --- | --- | --- | --- |
| Organism | GenePept  accession no. | Name | pI | Induction | Yield  (mg L^-1^) |
| *Xanthamonas sacchari* | AJC47165.1 | *Xsa*EXLX1 | 6.82 | IPTG | 20.0 |
| *Xanthamonas vasicola* | WP_010376099.1 | *Xva*EXLX1 | 7.88 | IPTG | 1.2-1.5 |
| *Dickeya aquatica* | SLM63089.1 | *Daq*EXLX1 | 8.42 | IPTG | 6.0 |
| *Dickeya solani* | AUC40652.1 | *Dso*EXLX1 | 6.16 | IPTG | 1.2 |
| *Clavibacter michiganensis* | WP_015488949.1 | *Cmi*EXLX2 | 5.78 | IPTG | 24.0 |
| *Streptomyces sp.* | WP_234442800.1 | *Ssp*EXLX1 | 7.84 | IPTG | 0.5-0.8 |
| *Nothophytophthora sp.* | RLN20739.1 | *Nsp*EXLX1 | 8.50 | MeOH | 50.0 |
| *Phytophthora cactorum* | KAG2772387.1 | *Pca*EXLX1 | 8.30 | MeOH | 41.0 |
| - *E. coli* BL21 and *P. pastoris* X-33 strains were used for expression of bacterial and eukaryotic expansins genes, respectively. - *E. coli* and *P. pastoris* expression hosts were induced with 0.2 - 1% IPTG and 3.0% methanol, respectively. | | | | | |

| **Table S3. Sugars analysis by LC-MS**. Soluble sugars (Glc-Clc4, mg/L) analysed released in treatment of the hardwood pulp with LPMO *Tr*AA9A alone (*Tr*AA9A) and in combination with BSA (BSA) or different expansins (*Xsa*EXLX1, *Daq*EXLX1, *Cmi*EXLX2, *Nsp*EXLX1, and *Pca*EXLX1) for 72 h. No oxidized monosaccharides were detected. | | | | | | | |  |
| --- | --- | --- | --- | --- | --- | --- | --- | --- |
| **Protein/Sugar** | | ***Tr*AA9A** | **BSA** | ***Daq*EXLX1** | ***Cmi*EXLX2** | ***Xsa*EXLX1** | ***Pca*EXLX1** | ***Nsp*EXLX1** |
| **Glc** | | 2.22±0.2 | 2.44±0.33 | 2.06±0.17 | 2.15±0.11 | 1.99±0.12 | 1.61±0.27 | 2.44±0.19 |
| **Glc2** | | 2.72±0.28 | 2.62±0.45 | 3.75±0.22 | 3.37±0.27 | 2.86±0.15 | 3.04±0.30 | 4.19±0.41 |
| **Glc2-diolC4** | | 6.85±0.64 | 6.52±0.38 | 9.28±0.56 | 5.87±0.63 | 6.66±1.16 | 7.64±1.05 | 11.14±1.89 |
| **Glc2-AldA** | | 0.71±0.05 | 0.60±0.07 | 0.89±0.05 | 1.18±0.07 | 0.70±0.06 | 0.70±0.09 | 1.02±0.09 |
| **Glc2-KetoneC4** | | 5.68±0.51 | 5.73±0.19 | 7.95±0.57 | 6.45±0.7 | 6.06±0.57 | 6.21±0.83 | 9.03±1.37 |
| **Glc2-DoubleOx** | | 0.27±0.02 | 0.22±0.02 | 0.29±0.02 | <0.05 | 0.23±0.02 | 0.25±0.03 | 0.32±0.03 |
| **Glc3** | | 4.50±0.33 | 5.02±0.48 | 6.94±0.24 | 7.05±0.55 | 5.58±0.22 | 6.23±0.48 | 6.53±0.50 |
| **Glc3-diolC4** | | 4.09±±0.19 | 5.72±0.93 | 8.11±0.50 | 7.56±1.04 | 6.45±0.23 | 7.06±0.86 | 7.31±0.95 |
| **Glc3-AldA** | | <0.05 | <0.05 | <0.05 | <0.05 | <0.05 | <0.05 | <0.05 |
| **Glc3-KetoneC4** | | 3.72±0.14 | 5.33±0.97 | 7.95±0.47 | 8.17±1.11 | 5.82±0.04 | 6.66±0.82 | 6.98±0.96 |
| **Glc3-DoubleOx** | | 0.05±0.01 | 0.11±0.07 | 0.13±0.02 | 0.15±0.05 | <0.05 | <0.05 | <0.05 |
| **Glc4** | | 1.54±0.13 | 1.59±0.14 | 2.37±0.55 | 5.01±0.27 | 1.91±0.12 | 2.60±0.30 | 2.48±0.46 |
| **Glc4-diolC4** | | 2.59±0.31 | 2.85±0.33 | 4.42±0.76 | 7.16±0.76 | 3.51±0.10 | 3.80±0.69 | 4.11±0.68 |
| **Glc4-AldA** | | <0.1 | <0.1 | <0.1 | <0.1 | <0.1 | <0.1 | <0.1 |
| **Glc4-KetoneC4** | | 1.87±0.24 | 2.06±0.18 | 3.42±0.21 | 6.37±0.84 | 2.48±0.15 | 2.91±0.38 | 2.98±0.55 |
| **Glc4-DoubleOx** | | 0.22±0.01 | 0.24±0.01 | 0.34±0.07 | 0.54±0.05 | 0.30±0.03 | 0.32±0.05 | 0.32±0.04 |


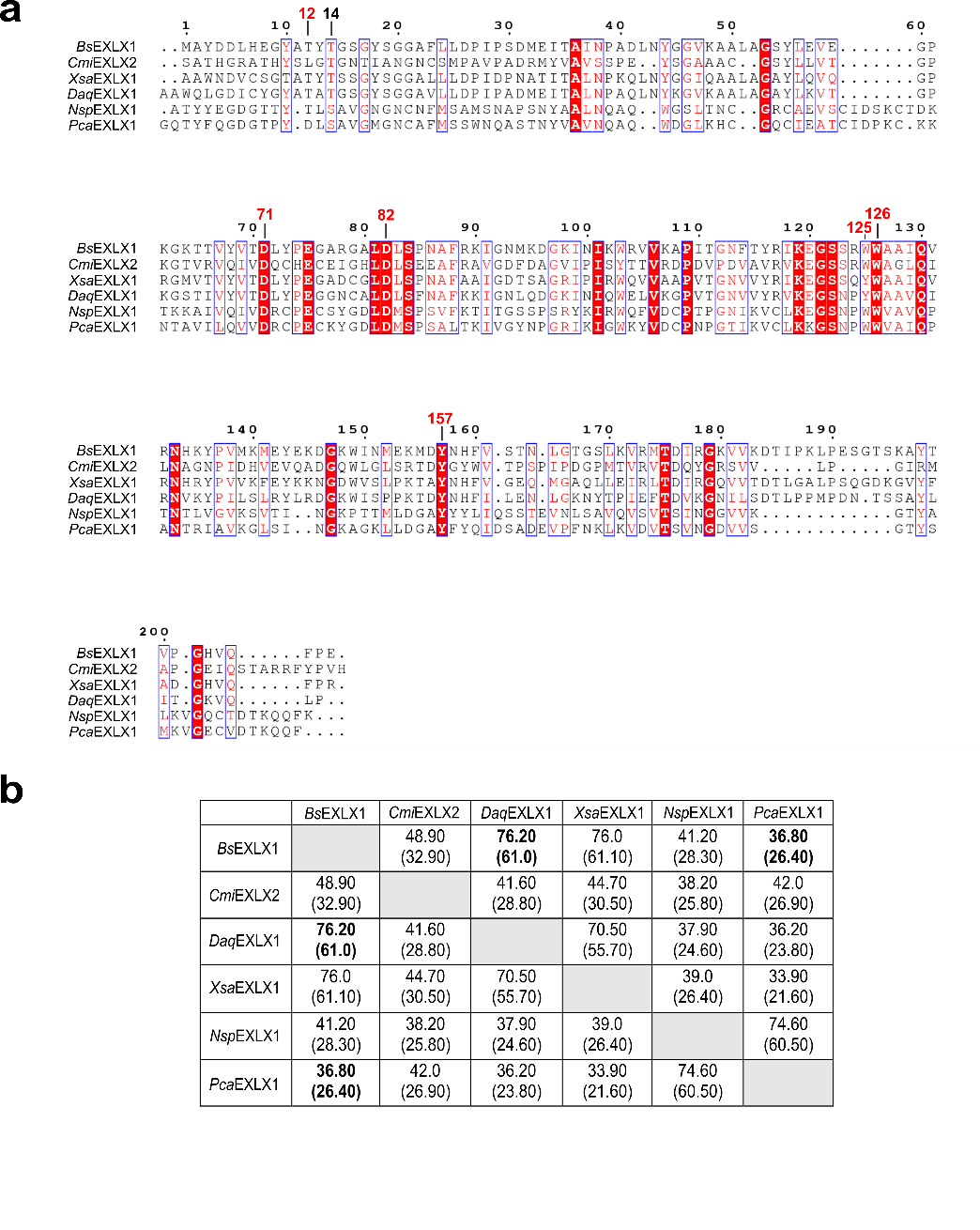


**Figure S1.** **Sequence similarity/identity among our targeted microbial expansins.** **a** Multiple sequence alignment using Clustal Omega of the five selected microbial expansins along with the primary amino acid sequence of the characterized protein from *Bacillus subtilis* (*Bs*EXLX1). The figure was generated using ESPript 3 with conserved amino acids highlighted in red font and strictly conserved residues highlighted in white font within a shaded red background. Numbering of amino acid residues are relative to alignment with *Bs*EXLX1. The predicted N-terminal signal sequences were truncated prior to sequence alignment. **b** Sequence similarity versus identity (in parentheses) between the microbial targeted proteins and *Bs*EXLX1. The most identical and distant sequences to *Bs*EXLX1 are highlighted in bold. The aligned sequences were from *Clavibacter michiganensis* (*Cmi*EXLX2; GenBank accession no: WP_015488949.1), *Xanthomonas sacchari* (*Xsa*EXLX1; GenBank accession no: WP_043094747.1), *Dickeya aquatica* (*Daq*EXLX1; GenBank accession no. SLM63089.1), *Nothophytophthora sp.* (*Nsp*EXLX1; GenBank accession no. RLN20739.1), and *Phytophthora cactorum* (*Pca*EXLX1; GenBank accession no. KAG2772387.1).


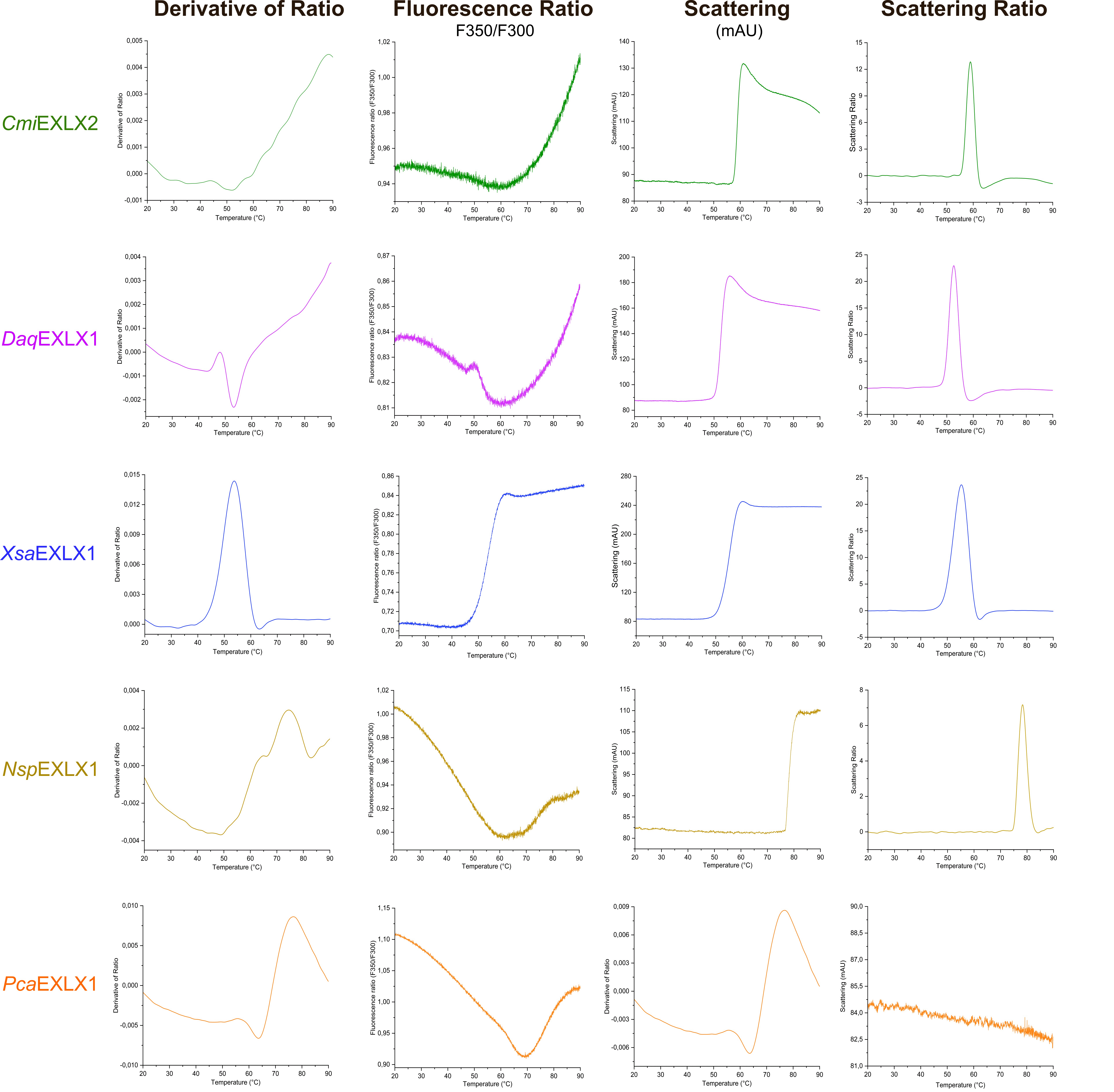


**Figure S2. Differential Scanning Fluorimetry (DSF) experiments.** Tm melting curves for five microbial expansins illustrating the data as Derivative of Ratio, Fluorescence Ratio (F350/F300), Scattering (mAU), and Scattering Ratio versus temperature (°C). All experiments were performed in 50 mM sodium acetate buffer, pH 5.5. The melting temperature Tm (°C) is defined as the inflection point of the melting curve. A shift in the Tm indicates a change in protein thermal stability with a decrease suggesting destabilization and an increase suggesting protein stabilization.

**
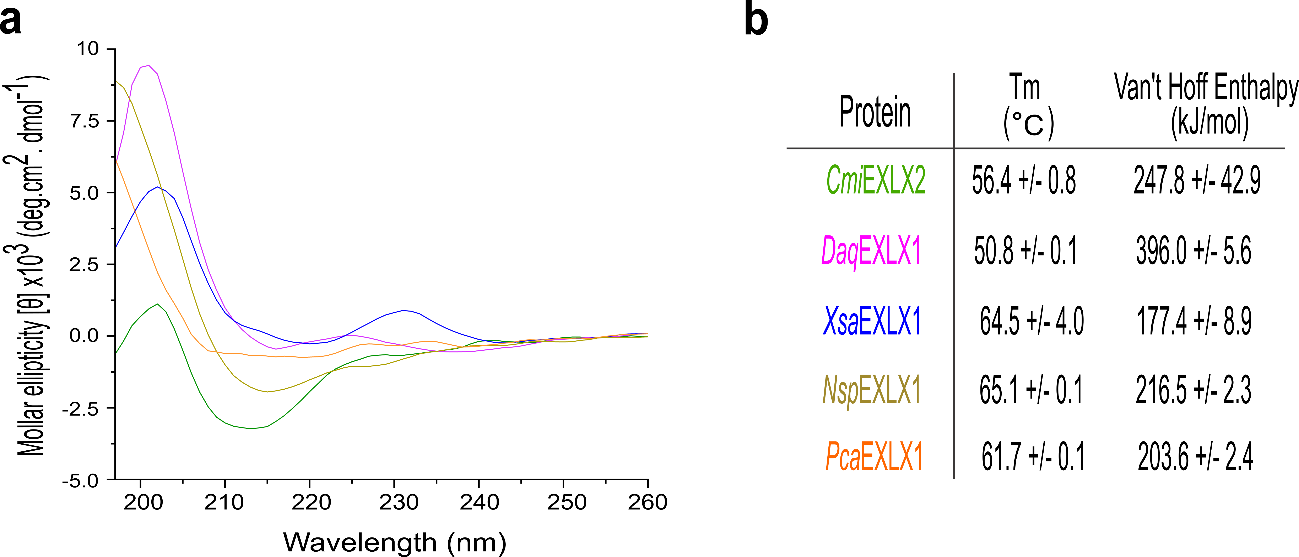
**

**Figure S3. Circular dichroism (CD) analysis. a** The CD steady-state wavelength spectra for the studied bacterial (*Cmi*EXLX2, *Daq*EXLX1 and *Xsa*EXLX1) and oomycete (*Nsp*EXLX1 and *Pca*EXLX1) expansins collected at 22 °C in 50 mM sodium acetate buffer, pH 5.5. **b** The calculated melting temperatures (Tm) and Van’t Hoff Enthalpies based on the analysis of the CD data.

**
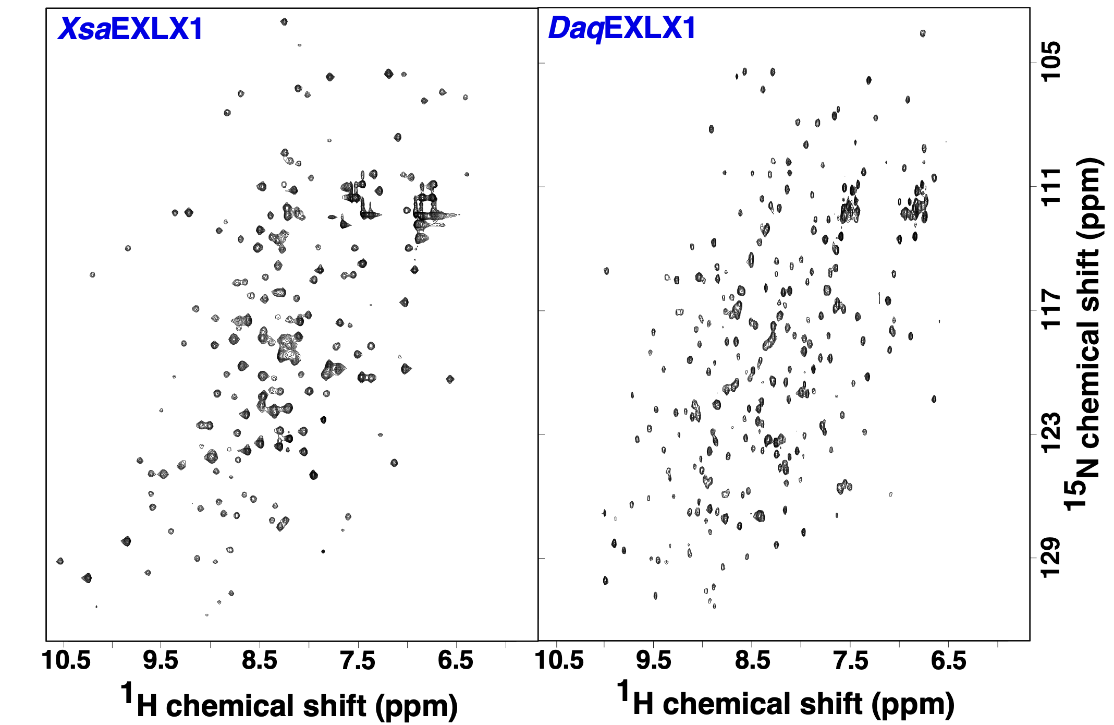
**

**Figure S4. NMR analysis of two bacterial expansins.** The ^1^H-^15^N HSQC spectra of ^15^N-labelled *Xsa*EXLX1 and *Daq*EXLX1 (~0.05 mM) collected at a proton resonance frequency of 600 MHz, 30 ºC, in 100 mM NaCl, 20 mM Tris, 1 mM DTT, pH 7.0. The wide chemical shift dispersion in both dimensions for both proteins is a characteristic feature of folded proteins.

**
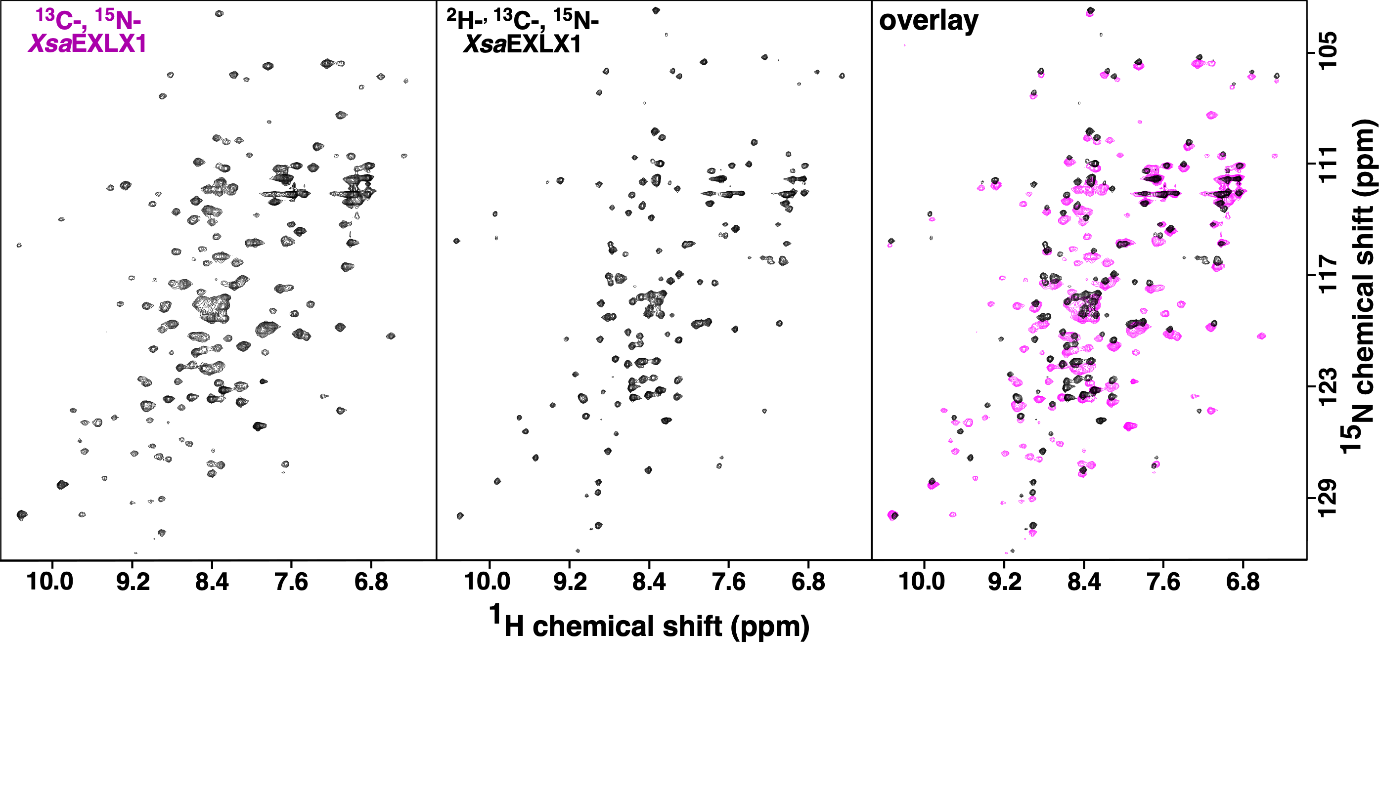
**

**Figure S5. Perdeuteration of *Xsa*EXLX1 increases intermediate (ms to ms) motion.** The ^1^H-^15^N HSQC spectra of ^13^C-, ^15^N-labelled (left) and ^2^H, ^13^C-, ^15^N-labelled (middle) *Xsa*EXLX1 (~0.05 mM) collected at a proton resonance frequency of 600 MHz, 20 ºC, in 100 mM NaCl, 20 mM Tris, 1 mM DTT, pH 7.0. An overlay of the two spectra is shown on the right (^13^C-, ^15^N-labelled (magenta) and ^2^H, ^13^C-, ^15^N-labelled (black) *Xsa*EXLX1). The only difference between the two spectra is the substitution of 98% D_2_O for H_2_O in the minimal media during the recombinant preparation of the samples. At this protein concentration, the fully protonated spectrum is missing ~40 expected amide resonances and an additional ~ 40 residues disappear after perdeuteration of the protein. The missing ~40 residues in the protonated spectrum suggests backbone regions of *Xsa*EXLX1 experience intermediate motion (ms to ms). The disappearance of an additional ~40 residues upon perdeuteration suggests these residues are close to the intermediate motion edge when protonated and the small increase in molecular weight due to perdeuteration is enough to shift more regions into the intermediate motion timescale.

**
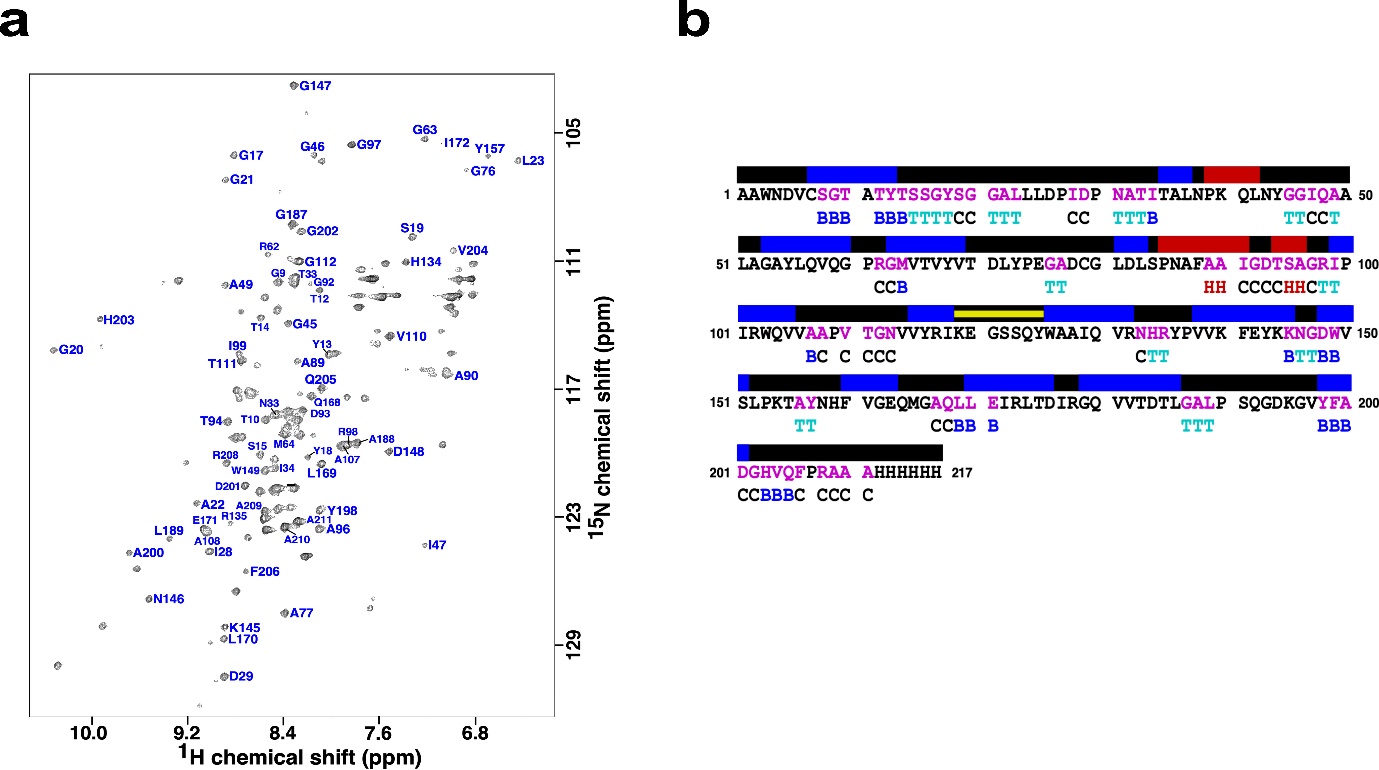
**

**Figure S6. Current chemical shift assignment for *Xsa*EXLX1 corroborates predicted Alphafold structure. a** The ^1^H-^15^N HSQC spectra of ^2^H, ^13^C-, ^15^N-labelled *Xsa*EXLX1 (~0.05 mM) collected at a proton resonance frequency of 600 MHz, 20 ºC, in 100 mM NaCl, 20 mM Tris, 1 mM DTT, pH 7.0, with the current amide assignments. **b** Primary amino acid sequence for *Xsa*EXLX1 with the assigned residues colored magenta. Above the sequence are the elements of α-helical (red) and β-strand (blue) secondary structure predicted by AlphaFold. Below the sequence are the elements of secondary structure determined from the analysis of the available chemical shifts (CSI 3.0; <http://csi3.wishartlab.com/cgi-bin/index.php>): a-helix = H, b-strand = B, turns = T, and random coil = C. The yellow bar denotes the linker between the two predicted domains of the protein. The residue numbered one is residue 30 in the full-length native protein (the signal sequence was removed in our construct).

**
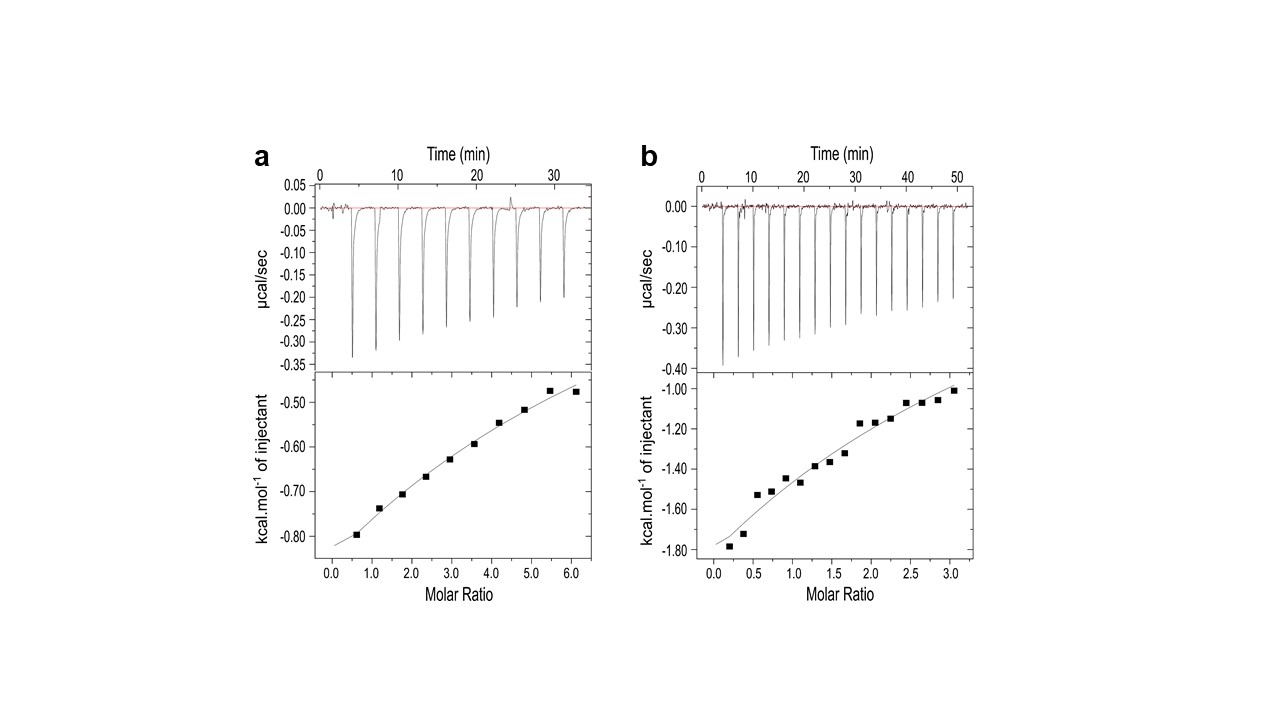
**

**Figure S7. Isothermal titration calorimetry (ITC).** The disassociation constant (*K_d_*) determined for the binding of **a** cellopentaose, 3.25 mM (DP5) and **b** xylotetraose, 1.12 mM (DP4) with *Cmi*EXLX2 determined using ITC at 25 °C in 50 mM sodium acetate buffer, pH 5.5. The upper and lower panel (in both **a** and **b**) illustrate the binding thermogram and the normalized integrated heat response (black squares) against the molar ratio of injected ligand to the protein concentration, respectively. The fits represent a one-site binding model.

**
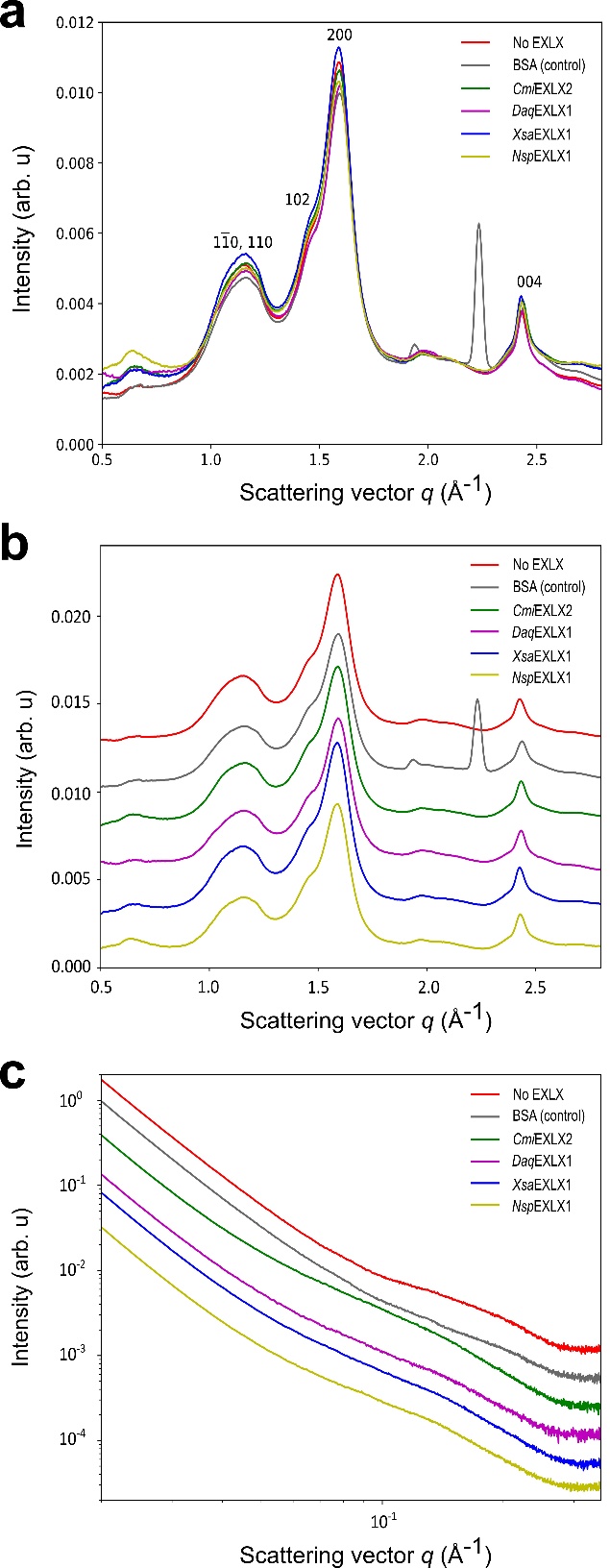
**

**Figure S8. X-ray scattering analysis.** The intensities of **a, b** wide-angle X-ray scattering (WAXS; vertically shifted for clarity in **b**) and **c** small-angle X-ray scattering (SAXS; vertically shifted for clarity) of treated hardwood (HW) Kraft pulp using four expansin-like proteins or BSA and a non-treated pulp sample (No EXLX). All data were generated in triplicate.


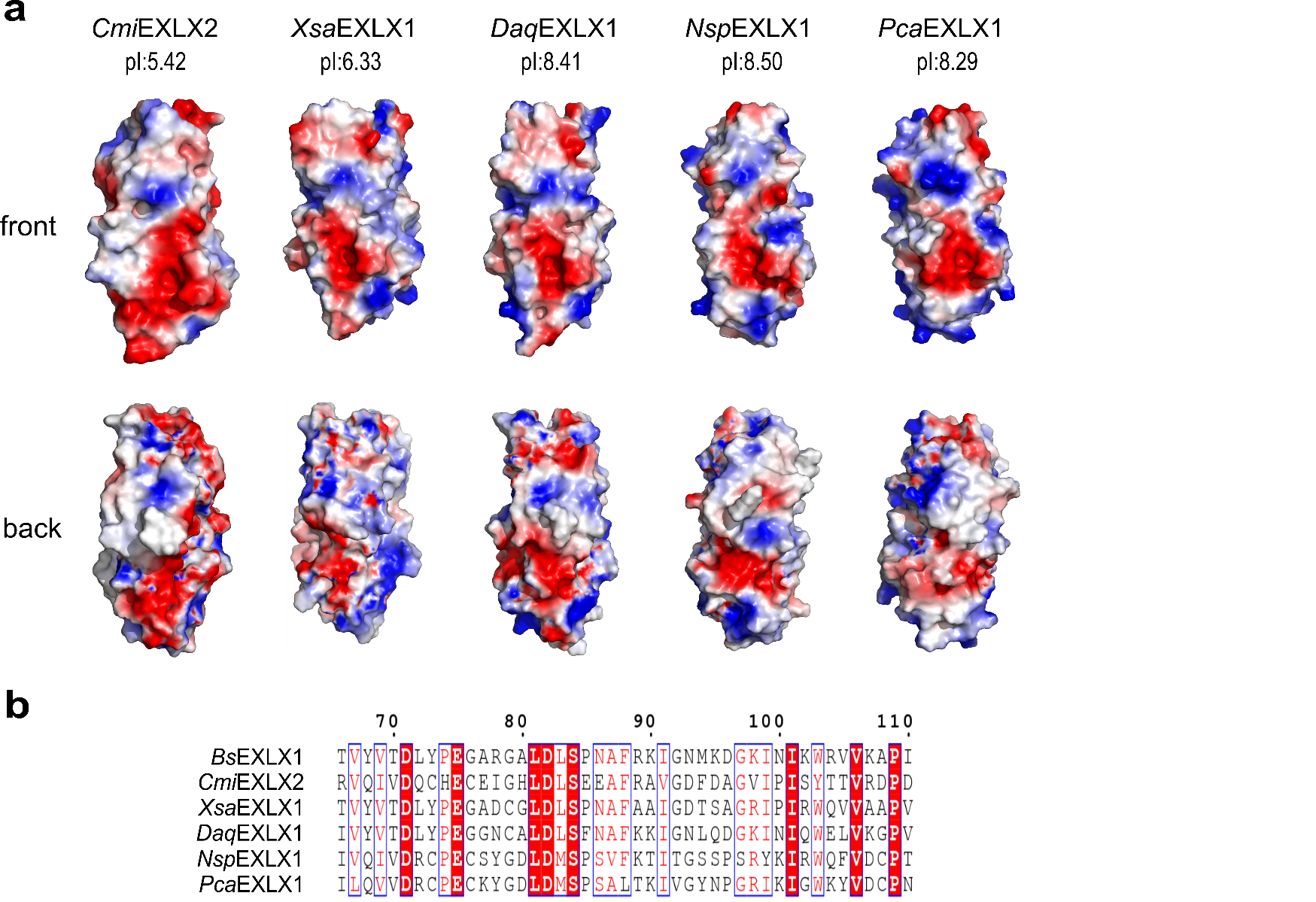


**Figure S9. Predicted electrostatic surface potentials of the targeted microbial expasins.** The models were prepared using AlphaFold available at EMBL-EBI ([https://www.ebi.ac.uk/Tools/sss/fasta/](https://eur01.safelinks.protection.outlook.com/?url=https%3A%2F%2Fwww.ebi.ac.uk%2FTools%2Fsss%2Ffasta%2F&data=05%7C01%7Cmajid.haddadmomeni%40aalto.fi%7C3c7e2e2a444a4fbf6d8708db4d3a5a0a%7Cae1a772440414462a6dc538cb199707e%7C1%7C0%7C638188685269673854%7CUnknown%7CTWFpbGZsb3d8eyJWIjoiMC4wLjAwMDAiLCJQIjoiV2luMzIiLCJBTiI6Ik1haWwiLCJXVCI6Mn0%3D%7C3000%7C%7C%7C&sdata=6pai5tR4Xi%2FlHsl6qMJtuAezRo9u9SJ6h6AIjTkJAo0%3D&reserved=0)). For each model the N-terminal signal sequence was removed and for *Cmi*EXLX2 the entire N-terminal appended CBM2 domain. The “front” models are orientated on the face of the polysaccharide binding site (PBS) with the N-terminal D1 domain underneath. The “back” models are a 180º rotation of the “front” face about the y-axis exposing the linker between the D1 and D2 domains. *Cmi*EXLX2 is a Gram-positive bacterium, *Xsa*EXLX1 and *Daq*EXLX1 are Gram-negative bacteria, and *Nsp*EXLX1 and *Pca*EXLX1 are eukaryotes. **b** Snapshot of the sequence alignment illustrating the different amino acid substitution in corresponding positions in *Cmi*EXLX2 (D1 domain).
